# Cenozoic Planktonic Marine Diatom Diversity and Correlation to Climate Change

**DOI:** 10.1371/journal.pone.0084857

**Published:** 2014-01-22

**Authors:** David Lazarus, John Barron, Johan Renaudie, Patrick Diver, Andreas Türke

**Affiliations:** 1 Museum für Naturkunde, Berlin, Germany; 2 United States Geological Survey, Menlo Park, California, United States of America; 3 Divdat Consulting, Wesley, Arkansas, United States of America; 4 Department of Geosciences, University of Bremen, Bremen, Germany; University of Vigo, Spain

## Abstract

Marine planktonic diatoms export carbon to the deep ocean, playing a key role in the global carbon cycle. Although commonly thought to have diversified over the Cenozoic as global oceans cooled, only two conflicting quantitative reconstructions exist, both from the Neptune deep-sea microfossil occurrences database. Total diversity shows Cenozoic increase but is sample size biased; conventional subsampling shows little net change. We calculate diversity from a separately compiled new diatom species range catalog, and recalculate Neptune subsampled-in-bin diversity using new methods to correct for increasing Cenozoic geographic endemism and decreasing Cenozoic evenness. We find coherent, substantial Cenozoic diversification in both datasets. Many living cold water species, including species important for export productivity, originate only in the latest Miocene or younger. We make a first quantitative comparison of diatom diversity to the global Cenozoic benthic ∂^18^O (climate) and carbon cycle records (∂^13^C, and 20-0 Ma pCO_2_). Warmer climates are strongly correlated with lower diatom diversity (raw: rho = .92, p<.001; detrended, r = .6, p = .01). Diatoms were 20% less diverse in the early late Miocene, when temperatures and pCO_2_ were only moderately higher than today. Diversity is strongly correlated to both ∂^13^C and pCO_2_ over the last 15 my (for both: r>.9, detrended r>.6, all p<.001), but only weakly over the earlier Cenozoic, suggesting increasingly strong linkage of diatom and climate evolution in the Neogene. Our results suggest that many living marine planktonic diatom species may be at risk of extinction in future warm oceans, with an unknown but potentially substantial negative impact on the ocean biologic pump and oceanic carbon sequestration. We cannot however extrapolate our my-scale correlations with generic climate proxies to anthropogenic time-scales of warming without additional species-specific information on proximate ecologic controls.

## Introduction

Marine planktonic diatoms (hereafter ‘diatoms’) are major components of the phytoplankton and are most common in regions of high productivity (upwelling zones) and in high latitudes [1]–[3]. Diatoms are important for the carbon cycle, generating ca 20% of global primary productivity [4], and, are key components of the ocean carbon pump via rapid sinking of large cells and aggregates [5]. Diatoms are thought to have diversified over the Cenozoic, and their evolutionary history is of great interest. Paleoceanographic studies frequently examine the role changing abundances of diatoms have had on the evolution of ocean environments and the carbon cycle. While studies may sometimes make use of quantitative estimates of past diatom export productivity from measurements of sedimentary opal abundance, over longer time periods and global scales, sedimentary opal abundance estimates are not available, and recourse is often made to diatom diversity (diversity here means species richness) as a proxy for diatom ecologic significance and export productivity (e.g. [6], [7]). The Cenozoic history of diatom diversity is thus of interest, not only to understand processes of evolution in plankton, but also for its use as a proxy for Cenozoic diatom ecologic influence and export productivity. How diatoms respond to future global warming (2–4° warmer by 2100 [8]) is also of considerable interest [3]. Most studies have used the living flora and focus on changing biogeography or ecosystem function [2], [9]–[12]. These studies have generally concluded that global warming, by reducing global latitudinal wind stress, will lead to more highly stratified, oligotrophic oceans, reduced abundances of diatoms, and possibly reduce the effectiveness of the ocean carbon pump. Studies of extinction risk in marine biota by contrast have concentrated on benthos or nekton: there are essentially no studies of marine plankton extinction risk.

**Figure 1:**
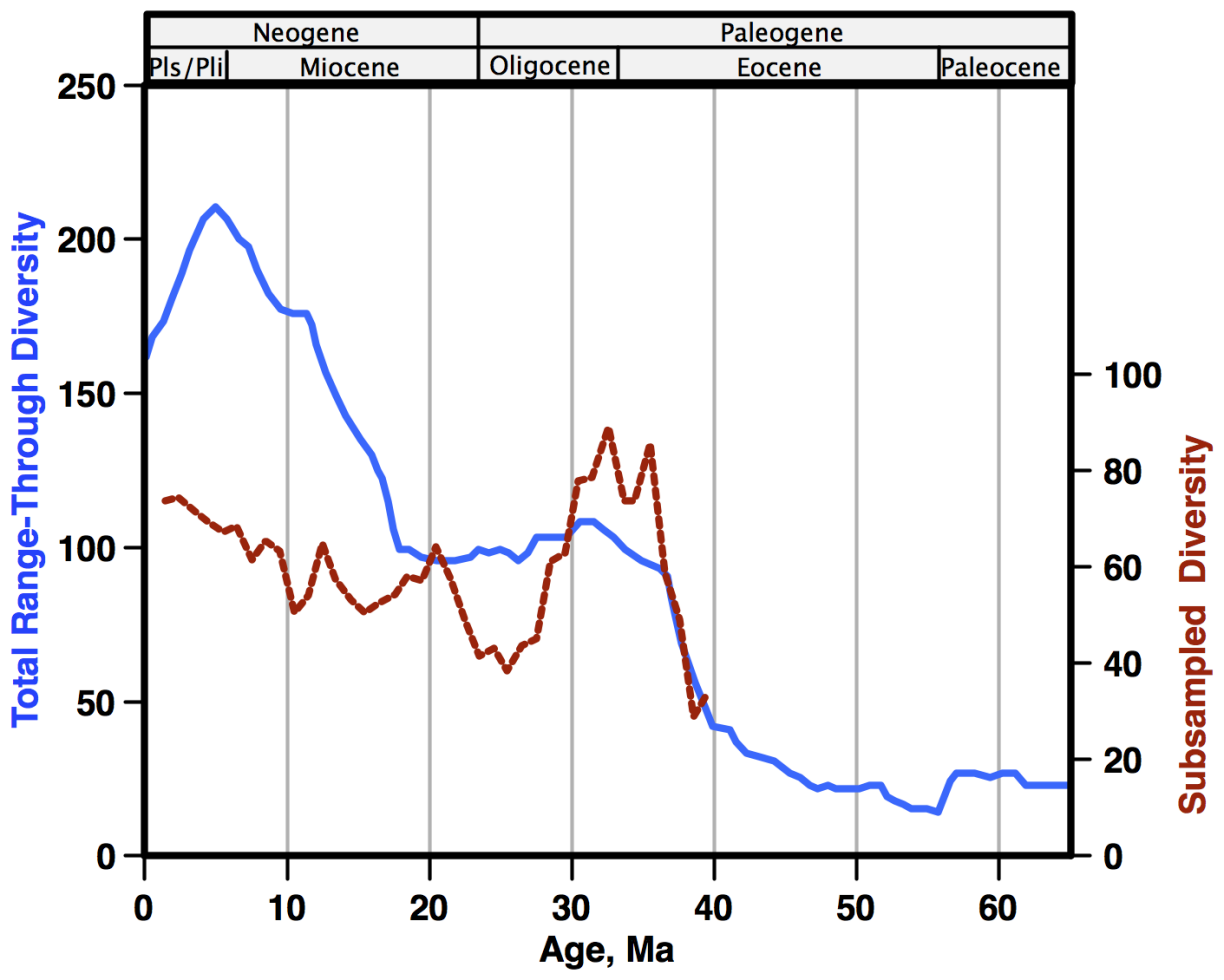
Published Cenozoic diatom diversity estimates, both from the Neptune database. Solid blue line - Spencer-Cervato (1) based on range-through simple (not subsampled) diversity; dashed red line - Rabosky and Sorhannus (4) based on rarefaction subsampling. Age scale: [42].

Diatom fossils in Cenozoic pelagic sediments [1], [13], [14] provide in principle an unusually good record of diversity [15]. However, although there have long been qualitative statements about diatom diversification over the Cenozoic in the literature, the first quantitative species-level assessment was only made by Spencer-Cervato [16] (Fig. 1), as part of the first systematic analyses of the Neptune database [17]. Spencer-Cervato calculated range-through diversity, which compensates for uneven data quality in individual time-bins, but which is sensitive to data outliers [15]. She evaluated the data for taxonomic problems (synonyms, etc) and eliminated the bulk of outliers by removing data adjacent to hiatuses in the database's age model library. Although she considered the effects of differing data amounts on diversity and calculated a simple ratio of diversity vs number of sections, a full analysis using standard diversity/subsampling theory was not attempted. All subsequent citations of her diatom diversity estimate use her simple range-through curve, not adjusted to number of sections. Rabosky and Sorhannus [18] (Fig. 1) later made use of subsampling algorithms, derived from ecology and newly popularized by their use in studying Phanerozoic fossil diversity, to provide a better estimate of diatom diversification after adjustment for differences in sample sizes over time. Uneven sampling intensity can bias observed relative diversity in comparisons between samples [19], [20]. Although aspects of Rabosky and Sorhannus' study are not documented (age scale, taxonomy, outlier detection and removal) the subsampling procedures employed are clearly defined: simple rarefaction, and three variations of sampling by lists (a ‘list’ = a ‘sample’, in the micropaleontologic terminology of this current study) and all gave very similar results. Subsampling does not give absolute but only relative values, ranging from zero to (at maximum) the arbitrary number of individuals subsampled, so a reference level is needed to compare curves. In Fig. 1 we have adjusted the scale of Rabosky-Sorhannus' simple rarefaction curve so that the Paleogene levels, on average, match those of the Spencer-Cervato curve. This makes the two main differences between the two analyses apparent. The Rabosky-Sorhannus curve shows a substantial decline in diversity during the late Oligocene-early Miocene, a period of gradual global warming, while the Spencer-Cervato curve shows nearly no change. The Spencer-Cervato curve displays a strong increase in diversity in the Neogene, a period of strong global cooling, relative to the Paleogene; the Rabosky-Sorhannus curve does not. These differences are critical to understanding how diatom diversity has responded to past global temperature changes, how changing marine export productivity is linked to changes in the global carbon cycle and changes in silicate weathering on land, and how these systems may respond to future global warming.

While diversity estimates compiled from raw data can have major data size biases, subsampling can also easily produce misleading results when underlying assumptions are violated (e.g., homogeneity of geographic structure and constancy of evenness of occurrence of species in samples), producing in such cases artificially ‘flattened’ diversity vs time curves. The Rabosky and Sorhannus results have already been questioned for this reason [21]. A new, robust estimate of Cenozoic diatom diversity history is thus needed, which takes into account sample size biases in occurrence data, the effects of data outliers, and potential biases due to changing geography and evenness. Ideally, alternate approaches to diversity estimation, such as catalogs [15] should also be considered. Deriving such a diversity history is the primary goal and result of this paper. In addition, we explore how our new diversity history correlates to environmental parameters such as paleoceanographic change, climate, carbon cycle and marine export productivity as derived from sedimentary opal. In these comparisons we do not attempt full analyses of possible causal mechanisms, as these are generally complex and require consideration of many factors, often with detailed time-series analysis and/or modeling, which is beyond the scope of our current study. We hope however by examining these correlations to point out possible important relationships between the evolutionary development of Cenozoic diatoms and environments, and thereby to stimulate further research.

## Methods

Our analytic strategy to estimate diatom diversity history is two-fold. First, we re-analyze the Neptune data. In contrast to prior studies, we explicitly test for sample size bias, and correct subsampled diversity estimates for both changing geographic endemism and changing mean evenness. Second, we use the complementary nature of catalog-derived diversity estimates [15] to confirm the robustness of our results using a new separate catalog (‘BDC’) of diatom species ranges by J. Barron. We also particularly consider the history of living taxa as most relevant to future responses to global warming, as extinct taxa may have had different biologic responses.

To test the sensitivity of diatom diversity to climate state, the resultant diversity estimates are compared to the global compilations of Cenozoic marine benthic foraminiferal isotope data for ∂^18^O and ∂^13^C of Zachos et al. [22], and to the Cenozoic record of biogenic opal in marine sediments [23]–[26]. Benthic ∂^18^O is an often used proxy for Cenozoic climate in studies of climate and evolution, e.g. [27]. This proxy reflects change in continental ice together with a strong high latitude/deep water ocean temperature signal [22], [28] and reflects many, for diatoms important, changes in the physical ocean environment that are correlated to changing polar/deep-sea temperatures over the Cenozoic (e.g. frontal systems, water column stratification, productivity). Benthic ∂^13^C reflects many different factors, but Cenozoic changes are usually interpreted as reflecting change in either the fraction of carbon sequestered in organic form into sediments, or changes in global organic isotope fractionation ratios (e.g., global increases in low C12-enriched plants: C4 grasses, diatoms) [29], [30]. We also compare diatom diversity history directly to the paleo-atmospheric pCO2 estimate of van de Wal et al. [31] for the interval 0–20 Ma. While all paleo-atmospheric pCO2 estimates have uncertainties (for the dataset used, ca 20–40 ppm) [31], this estimate provides continuous, high resolution values with a consistent methodology, and thus suffers less from the variability in other compilations due to systematic differences between estimation methods, e.g. [32].

### Data sources and analytic tools

The Neptune database was originally developed in the early 1990s [17], initially analyzed in the late 1990s [16] and ported to the internet by the Chronos project (USA) in the early 2000s [33]. The Chronos version has for some years now been in a somewhat unstable post-funding maintenance state, and one recent user [34] reported finding errors in the calcareous nannofossil content. Errors are important if they create outliers that extend ranges of species in time, causing incorrect diversity estimates. The NSB version of Neptune (for ‘Neptune Sandbox Berlin’) used in this study is a new implementation of the database hosted at the Museum für Naturkunde in Berlin. It was forked off the Chronos version of the Neptune database in early/mid 2010. Analyses of NSB content [35], [36] have specifically checked, and not found data errors like those reported by Lloyd et al. [34], suggesting that these, if present in Chronos Neptune, postdate the NSB fork. As a further check on possible data errors we apply a Pacman analysis [35] to examine the effect of outliers in our diatom data (see below). NSB is otherwise similar in content to the Chronos Neptune database. It contains data for individual species according to the original name published, plus synonym lists that allow data to be linked that were published under different names. The Neptune database is restricted to deep-sea drilling sources, and its age models and taxonomy are a mixture from numerous authors [16], [17]. For our study we used the built-in synonym information to extract all occurrence data for 662 valid species and their synonyms, for a total of 63,675 occurrence records.

The species range catalog (Barron Diatom Catalog, ‘BDC’, Table S1a) is a first and last occurrence database similar to others used in paleontology and micropaleontology for evolutionary analyses [27], [37]–[39]. This database is new and the first such compilation available for diatoms. It was compiled from a variety of literature sources (, total = 62), including the deep-sea drilling reports used to compile Neptune (ca half of the sources used), additional primary studies, many land sections (majority of the remainder), plus a few papers that themselves are syntheses of other literature. It uses a uniform taxonomy, and ranges are evaluated for various other errors e.g. [15]. It records 529 species' age ranges and their biogeography (tropical, North Pacific, Southern Ocean). Species first and last occurrences were judged when possible by examination of the actual occurrence data and careful evaluation of the accuracy of the age information available for the occurrences, with the single best source being chosen to provide the age value for first or last occurrence. This is admittedly more subjective but is much better at identifying and filtering out questionable data than a purely automatic computerized procedure, and is the data compilation method frequently used in other micropaleontologic studies, e.g. [27].

A small number (ca 10%) of the taxa in the BDC have ranges determined in part from the Chronos Neptune database. The two data sources are thus not 100% independent of each other. However virtually all taxa present in the BDC are present as well in the primary literature used in the compilation, so use of NSB at most provides a minor adjustment to the range of a small number of species rather than significantly affecting diversity itself.

### Data errors and choice of time bin size

All marine microfossil diversity data has potential sources of error [15]. These include uneven completeness of diversity recording by different authors, non-uniform data coverage by time, geographic region and author, differing amounts of fixed-list biostratigraphic vs diversity survey data, age model errors, reworking, and others. Diversity data is drawn for example from a much broader number of deep-sea sections, with on average poorer quality age models, than the much smaller number of sites with high-quality age models typically used for high-resolution paleoceanography, and compilation of stable isotope curves. The complexity of the sources of error precludes formal analysis, but based on our own experience, we feel that binning such diverse global diversity data much below .5 my or 1 my is unlikely to bring much improved real resolution. Age model mismatches are mostly <.5 my but can sometimes be >0.5 my or even 1 my, particularly between low and high latitude sites [40], [41], while smaller bins both increase random effects of other aspects of data quality due to smaller data pools, and increase the number of bins with too little data in subsampling procedures. Larger bins are not desirable, decreasing our ability to compare more rapid (often significantly less than 1 my) changes in climate state to diversity. In smaller e.g. regional, more homogeneous microfossil diversity data sets Renaudie and Lazarus [36] explored the effect of bin size on diversity studies and find no significant effect between 0.5 and 1 my bins. Their study also points out that the incompleteness and/or biostratigraphic bias of data available in databases such as NSB also limits effective resolution of time-series change in primary biodiversity signals to at best ca .5 my. We therefore analyzed our data using .5 and 1 my bins. Comparisons of selected identical analyses run at these two different resolutions (not presented) showed no significant differences.

### Age scale and chronology

The large majority of the data in our study, including the NSB data and most published literature data was originally calibrated to the Berggren et al. 1995 timescale [42], including all NSB data and stable isotope environmental data. Only the BDC ranges were in the more recent Gradstein et al. 2004 timescale [43]. For this study some early analyses exploring the nature of sampling bias, or comparison to published literature, were carried out using the older Berggren scale. For later analyses, including all comparisons of diversity to environmental parameters, the Gradstein et al. scale was used. Differences between scales are mostly <1 my and have no significant effect on the results of the study.

### Subsampling

As biotas usually consist mostly of rare species, a sample's diversity is usually much less than the total diversity of the sampled biota; thus sampled diversity increases with sample size. If sample size varies in a data series, sampled diversities, and also range-through diversity from catalogs compiled from such samples, can be biased [19]. Standardized subsampled Neptune data is needed to correct for the strong variation of sample sizes in Neptune with time. Subsampling though yields biased results if either taxa ranked abundance distributions are not constant between samples; or if clustering of taxa by region change. Both of these phenomena occur in Neptune Cenozoic diatom data. A variety of subsampling algorithms have been proposed, including sampling a fixed number of individuals (simple rarefaction) or sampling a fixed or variable number of samples (lists, in subsampling terminology). We use both sampled-in-bin classical rarefaction, and a new method, SQS [44]. Subsampling by list was not used in our study for several reasons. When the average number of species in a list is short compared to the total diversity in a bin, which is true of NSB data [15] the effectiveness of list methods is reduced (and collapses as list size approaches 1 taxon to simple rarefaction); modeling subsampling by lists is more complex and requires information on taxon clustering in lists (e.g. samples), which would make modeling the effect of evenness on subsampling (below) much more difficult; the newest SQS subsampling algorithm [44] is based on (size adjusted) simple rarefaction, making it easier to compare results by using (our own alternate size adjusted-see below) simple rarefaction in our analysis; lastly, in the prior study by Rabosky and Sorhannus [18], simple rarefaction and list type analyses of Neptune data gave very similar results.

### Geographic correction

The SQS algorithm was specifically developed to compensate for the inability of classical subsampling methods to adequately capture change in diversity associated with the development of geographic differences in distributions (e.g. endemism) [44]. Rather than taking a fixed number of occurrences, SQS varies the sample size to compensate for diversity underestimation due to increased geographic structure [44]. As the SQS method is still very new, we use as well an alternate method to correct for changing endemism, making use of the well resolved geographic affinities information for each species in the BDC database. The ratio of the largely mutually endemic polar to tropical species in the BDC data for each time interval is used to correct a simple classical rarefaction estimate of diversity for each time bin, as the geographic diversity ratio in time bins in the BDC should not be sample size biased; the resultant curve is our ‘PTR’ sampling method.

### Evenness correction

**Figure 2:**
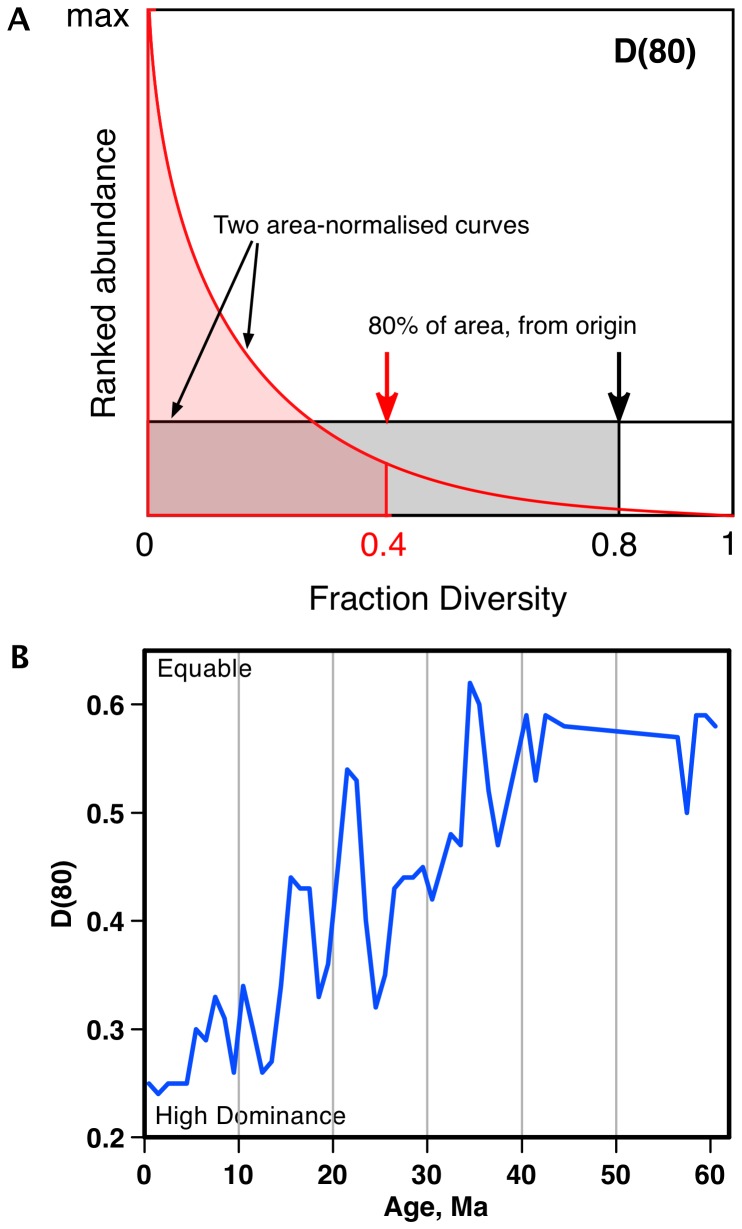
Equability (increasing dominance) in relative frequencies of diatom species in NSB occurrence data over the Cenozoic. a) Dimensionless shape metric D(80), defined as fraction of total diversity reached in ranked frequency occurrence data at 80% of total area. For data with all frequencies equal (flat black line) D(80) = 0.8; D(80) decreases with increasingly unequal frequencies. b) D(80) vs time in Cenozoic NSB diatom data. Age scale: [43].

To correct for the effect of changing evenness (degree of similarity in relative abundances of different species) over time, a simple correction factor was computed based on 1) a simple ranked abundance data shape metric ‘D(80)’ (Fig. 2a) that quantified the changes in evenness patterns in each time bin (Fig. 2b), and 2) a scaling of this metric according to the results of a simulation of the effects that different evenness patterns in the data have on rarefaction. The D(80) metric, here defined as the fraction of total diversity reached in ranked frequency occurrence data at 80% of the total area, is only one of any number that could be used, but is linear to cumulative frequency, is not weighted towards a few most common taxa, and is similar to the cumulative frequency adjustments used in SQS geographic correction. We quantified a major shift over time, from very even occurrence frequencies in the Paleogene, to highly uneven distributions in the Neogene (Fig. 2b).

**Figure 3:**
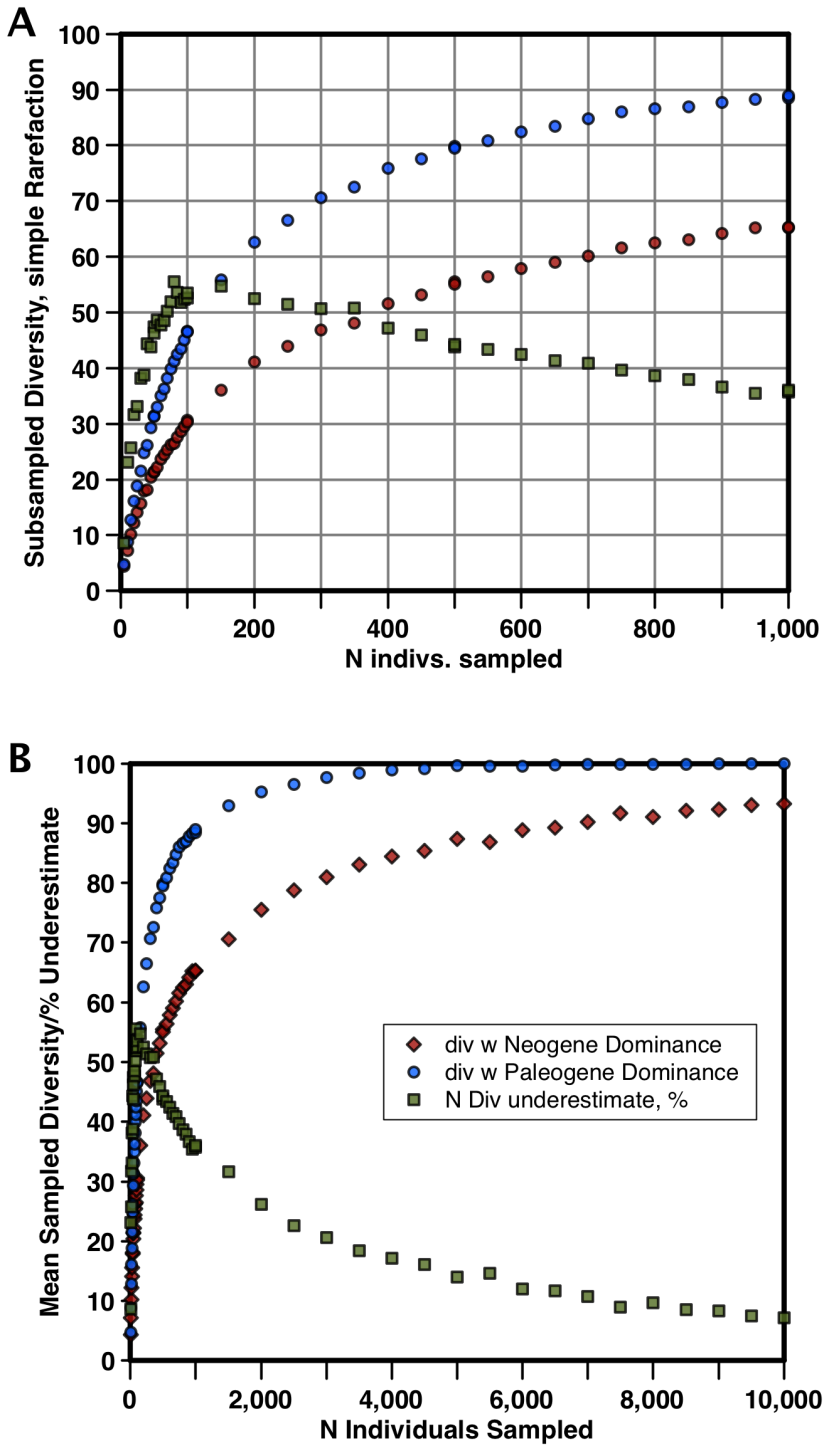
Results of simple rarefaction subsampling two virtual computer populations of equal diversity (100 taxa) and total sample size but with averaged Neogene or Paleogene relative abundance distributions as seen in NSB diatom occurrence data. a) for subsample sizes between 0–1,000; b) subsample sizes 0–10,000. Blue dots - Paleogene subsampled diversity; red - Neogene subsampled diversity; green squares - percentage underestimation of Neogene diversity relative to Paleogene diversity. Each point is an average of 50 subsampling trials. Age scale: [43].

The pooled data for species ranked occurrences used were from two major time intervals (44-31 Ma: Paleogene, and 14-1 Ma: Neogene). Each data set was scaled to the same total area ( = number of occurrences or total sample size) for the ranked abundance curves, interpolated down to the same number (100) of virtual species, and these two virtual data sets - having the same diversity and total sample size, differing only in the relative frequencies of taxa - were subsampled using simple rarefaction at a range of sample sizes. Each sample size was repeated 50 times and averaged. The mean diversity found by subsampling for a given subsample size in each of the two virtual populations was used to calculate the degree to which the diversity of the low evenness Neogene population was being underestimated compared to the Paleogene population. The results for a wide range of sample sizes are shown in Figs. 3a and b. For sample sizes within the range used in rarefaction subsampling of NSB, both in our study and in that of Rabosky and Sorhannus [18] (100 and 96 individuals, respectively) the correction factor needed to make the diversity obtained by rarefaction the same is ca 1.55. Given the mean D(80) values for these two pooled data sets (0.551 and 0.282 for respectively, Paleogene and Neogene), and no correction factor for the Paleogene pooled data (i.e. = 1) a linear equation giving the required correction factor (y) as a function of D(80) can easily be computed: y = 2.127–2.04*D(80). This correction function result was applied to both of our subsampled results using the bin specific D(80) evenness metric values (Fig. 3b) to correct for evenness effects. Note that we did not use the simpler method of evenness correction proposed by Alroy [44], of just leaving the most common species out: we found this very ineffective with the diatom data of our study. This is not surprising since differences in evenness are seen over a large fraction of the species in the diatom data, not just the very most abundant one.

### Outliers and other errors in occurrence data

**Figure 4:**
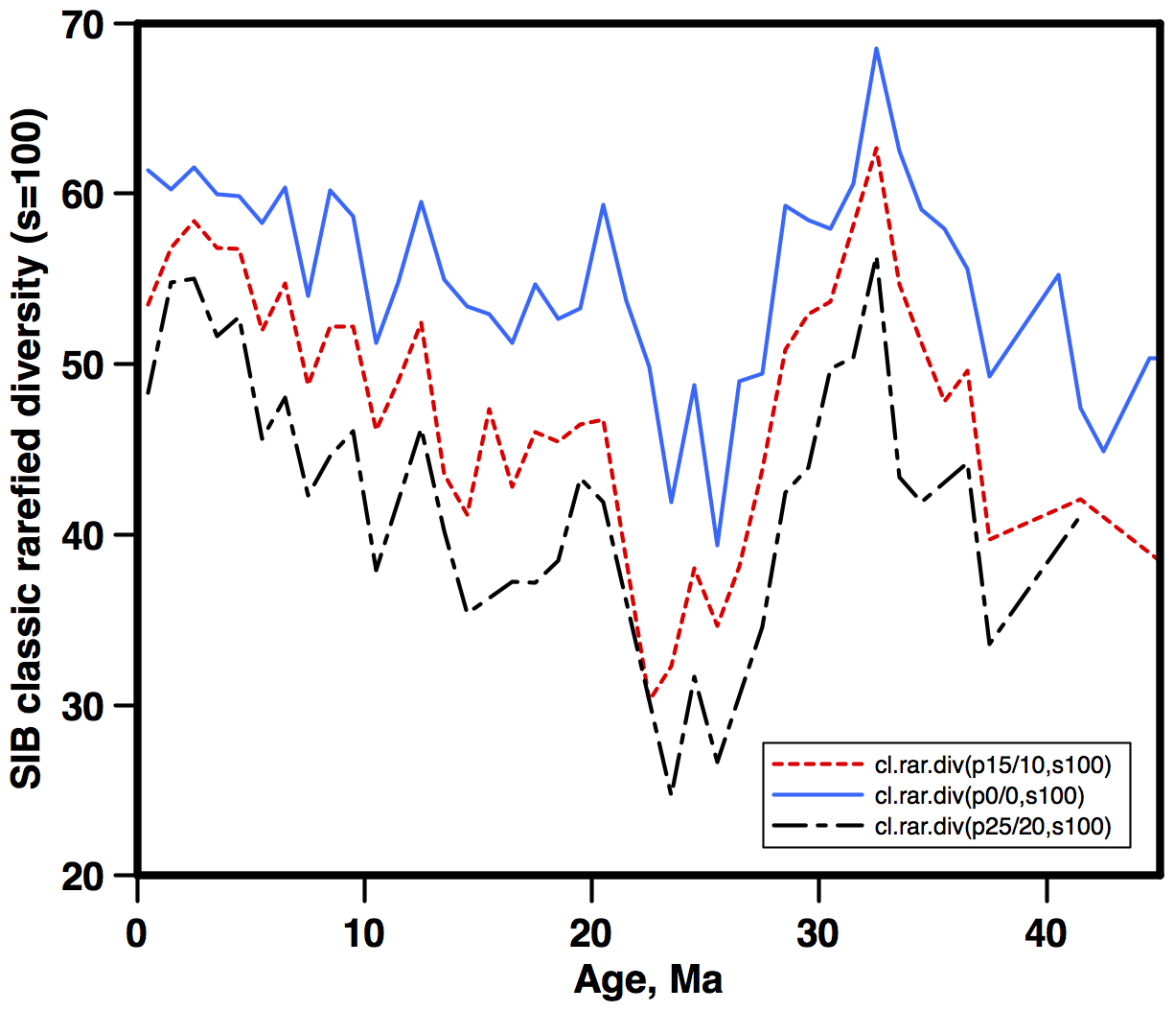
Subsampled diatom diversity (sample size = 100) from the NSB database, using different levels of Pacman trimming of data from the ends of species ranges (7) to remove outliers due to possible data errors in NSB. Blue solid line - no data trimming; dashed red - 15% of the youngest and 10% of the oldest occurrences for each species removed; alternate dashed black - 25% of the youngest and 20% of the oldest. Age scale: [43].

Outliers can have major effects on range-through calculations of diversity [15]. However, so long as they are distributed at random and are uncommon, they have little effect on subsampled diversity estimates. Outliers are an unavoidable aspect of large data compilations, and also exist in the NSB database due to intrinsic aspects of the data such as reworking, taxonomic mis-identifications and age model errors [15]. While trimming fixed percentages of the range ends of age-composited occurrence data (‘pacman trimming’) can remove most outliers [35] this procedure can also create edge effects, particularly for the youngest time bin(s), and thus complicate comparisons of diversity and environmental history in the late Neogene. For this reason we did not apply a Pacman trim in our main diversity reconstruction. We checked however for the possible effect of outliers on our sampled-in-bin diversity estimates by running successively stronger Pacman trims on the data and applying simple rarefaction to the output, examining the curves for changes in shape (Fig. 4). Even with very strong trim levels (25–45% of the total occurrence data, vs ca 10% in the study of Lazarus et al [35]) the basic rarefaction curve was largely unaltered, although the dynamics were stronger, particularly the relative increase in diversity over the Neogene. This may be an artifact of strong trim levels depressing diversity in bins where many taxa are jointly near the ends of their true ranges, e.g. a gradual turnover between relatively homogeneous late Paleogene vs early Neogene floras. This analysis shows that our ‘untrimmed’ subsampled NSB diversity curve is not strongly affected by outliers, and if anything is a conservative estimate of the degree of dynamics of diversification over the Neogene.

### Detrended analyses

In comparing time-series data, one problem occurs when both variables have a trend, resulting in a correlation being seen even if there is no causal relationship between them. A second problem arises when one variable cannot respond fully to changes in the forcing variable due to internal limits (e.g. stochastic effects), which will affect the observed correlation between variables. There is considerable a-priori evidence from diatom biology and biogeography to expect diatom diversity to respond to climate, and thus to expect the long-term as well as short term correlations to be at least in part causal, not chance. There is also no evidence in our diversity-climate comparisons for significant mis-correlation due to limitations in the freedom of response in the diatom diversity data: even short-term changes in climate state are partially mirrored by diatom diversity change, and maximum bin-to-bin step size changes in diatom diversity, both increasing and decreasing, match in amplitude those of the presumed climate forcing function. Such observations however cannot fully exclude the existence of correlation artifacts. One established method of testing for the robustness of a correlation is to use linear regression to detrend, or to first-difference the time series and compare only residuals. This has the major disadvantages of potentially removing one of the main (long-term) components of the signal, and by using only residuals, unless errors in individual values (noise) are low, a significant loss of ability to detect correlations, and thus correctly evaluate a hypothesis.

Given the existence of a variety of errors limiting individual bin estimates for diversity (described above under Data Sources) we do not feel that first-differencing the data would yield a time-series with a usable signal. Hannisdal [45], [46] for example has very recently proposed new methods for analysis of correlation between time series, but their robustness to different types of complex sources of data error (the methods use first differences residuals) is not yet, in our opinion, sufficiently documented to justify employing in our study. We believe that a somewhat less severe detrending by simple linear regression should retain a significant signal, and at least allow us to test for co-incidental correlation of unrelated primary trends. Detrended versions of all significant results were thus also analyzed for statistical significance, and the results of these tests are considered in our discussion and conclusions.

## Results

### Simple diversity vs sample size

**Figure 5:**
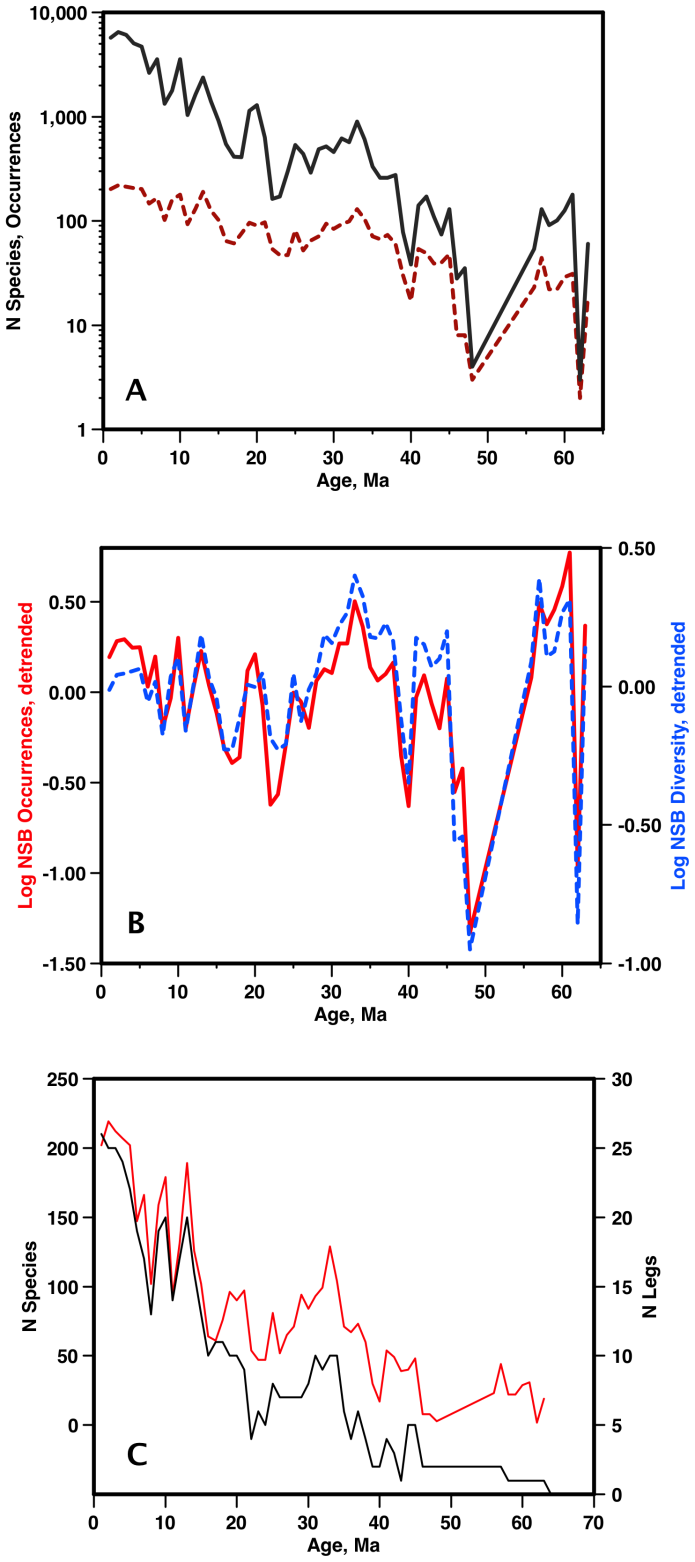
Neptune (NSB) diatom diversity vs data density. a) Diversity (red) vs number occurrences (black). Note log scale and different slopes. b) detrended NSB diversity (blue) vs number occurrences (red) (detrending by residuals of linear regressions vs. time). c) NSB total in-bin diversity (red) vs number of distinct drilling program source Legs (black). Due to DSDP/ODP/IODP staffing/publication policies, Legs are a very good proxy for number of papers. Age scale: [42].

Although the primary justification given by Rabosky and Sorhannus [18] for a new analysis of the Neptune data was the expected correlation of diversity to sample size, they surprisingly did not explicitly examine this effect. Here we provide an analysis of the correlation between simple diversity and sample size for both sources of data used in our study - the NSB version of the Neptune database, and the BDC listing. NSB occurrence data is strongly correlated to the number of samples in the time bin (Fig. 5a), although the relationship is not linear. The correlation is particularly pronounced in detrended data (Fig. 5b). Data compilations such as the BDC are done at the level of publications, and underlying correlations to sample size could in theory be masked. To see if this is true for the micropaleontologic literature, we compared simple NSB diversity per bin to the number of Legs (ca 2-month long individual DSDP/ODP expeditions) with data in that bin. Due to long standing policies on leg staffing and sample access, the large majority of deep-sea drilling legs have only a single primary diatom paper reporting occurrence data. There is still a strong correlation between number of legs/bin and diversity/bin (Fig. 5c), suggesting that using numbers of papers as a proxy for number of samples does not mask a sample size-diversity correlation.

**Figure 6:**
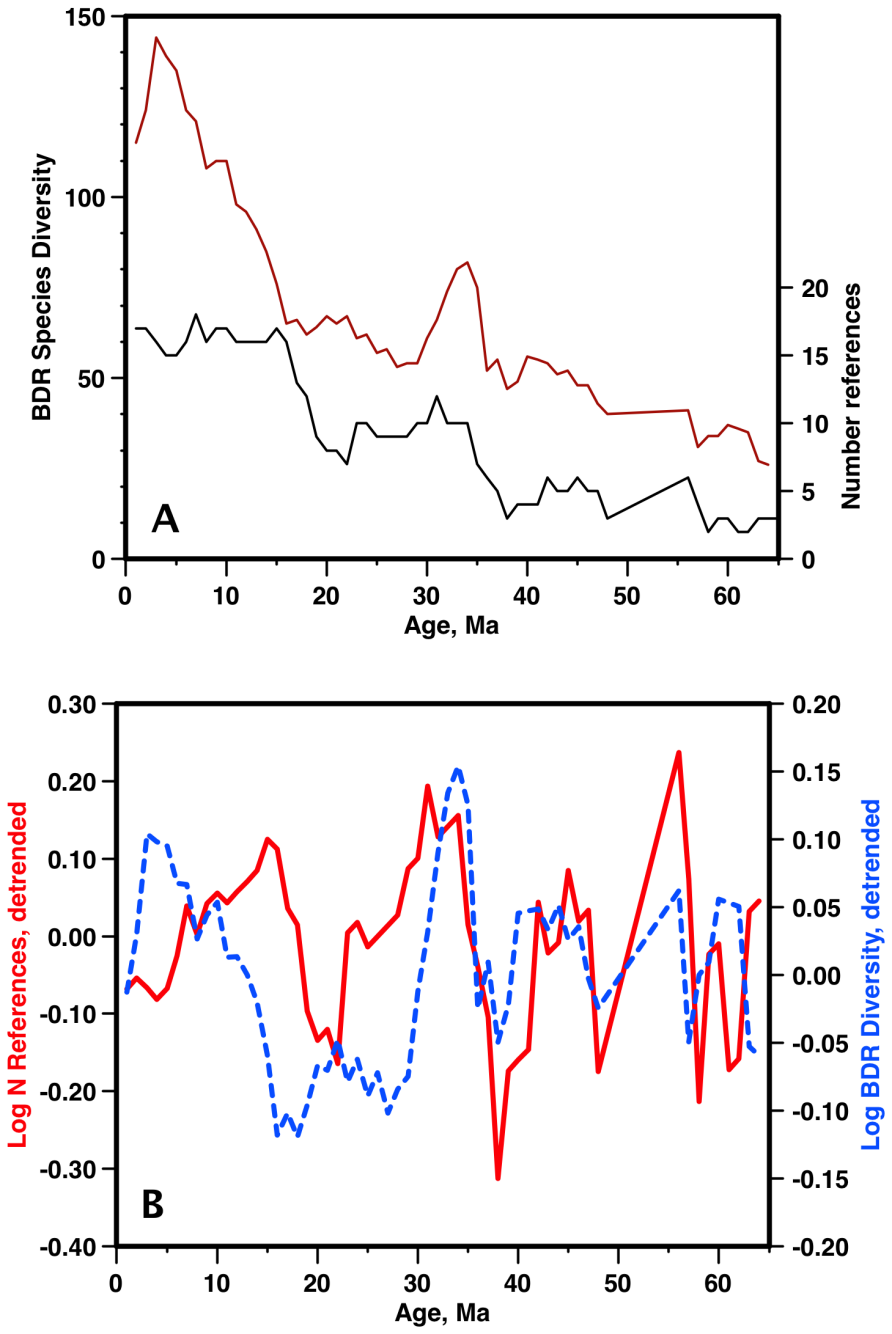
BDC diversity vs data density. a) BDC diversity (red) vs number of sources (black - papers or other as given in table ST1b). b) detrended BDC diversity (blue) vs number of sources (red). Note lack of correlation in 0–30 Ma interval. Age scale: [42].

By contrast, similar analysis shows that there is only a weak overall correlation between the BDC diversity and the number of papers used in its compilation (Figs. 6a and 6b). Most importantly, there is no discernible correlation between numbers of papers used and diversity in the Neogene, either in the raw or the detrended data, and raw BDC data may thus provide (vs. raw Neptune) a less biased estimate of diversity, particularly in the Neogene.

In both data sources it is also clear that the quantity of data declines with time, and in particular, very little data is available to estimate diatom diversity below ca. 40 Ma (late middle Eocene), and particularly between ca 42–55 Ma.

### Diversity history of diatoms

**Figure 7:**
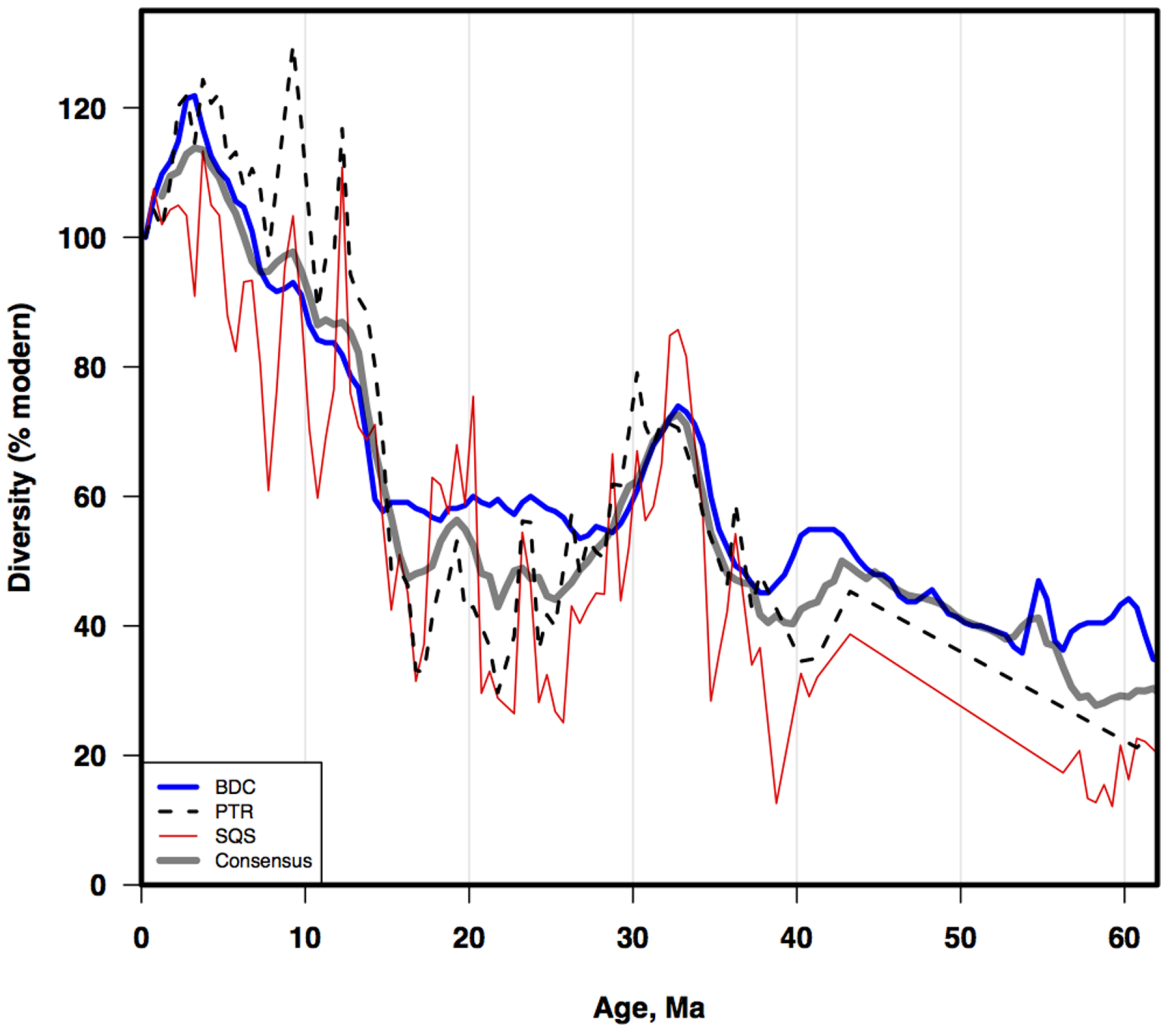
Cenozoic diatom diversity. BDC - total range-through diversity from Barron diatom catalog; PTR - evenness corrected subsampled NSB diversity with geographic correction computed from polar-tropical diversity ratio in BDC; SQS - evenness corrected diversity using SQS subsampling. Percent scale (right) from BDC values. Age scale: [43].

**Figure 8:**
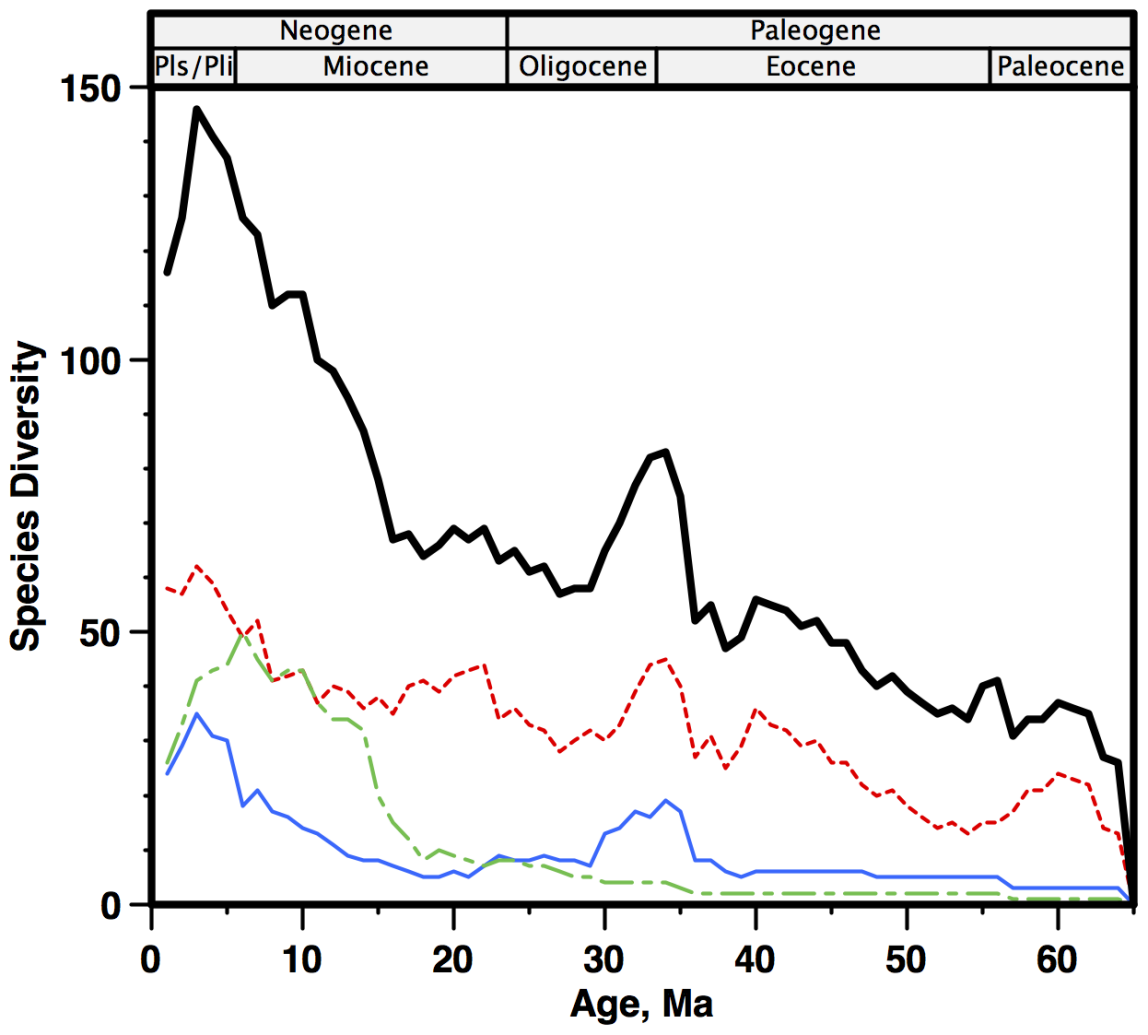
Cenozoic global and regional diatom diversity (range-through) from the BDC catalog. Bold solid black - total; red dash - tropical; green alternate dash - North Pacific; solid blue - Southern Ocean. Age scale: [42].

Our analyses yielded three different estimates of Cenozoic diatom diversity, using different methods and different data sources: the SQS and PTR estimates from the NSB database, and the BDC estimate from the range catalog (Fig. 7). For comparison purposes all diversity results are z-score normalized, as SQS/PTR yield relative change only. Cenozoic BDC range-through diversity (total: curve ‘BDC’; and by region, Fig. 8) increases strongly toward the present, with a transient late Eocene peak; and a larger, rapid Neogene-Recent rise (ca 15-0 Ma), mostly from developing endemic polar floras (Fig. 8; [13], [14]). The NSB estimates, despite occasional implausibly high rates of change over short time intervals, are very similar to the BDC estimate. All three estimates are remarkably similar for the Neogene, showing rapid increase 15-0 Ma. Given this similarity, we use the average of the three results as our new Cenozoic diatom diversity estimate (‘LBR’). The LBR average reflects the combined common signal from three different methods/datasets, while the average removes much of the discrepancies (noise) found only in a single estimate. Below ca 42 Ma the LBR estimate is not very robust, as for the most part, too few data were available in the NSB database to allow estimates of diversity using the SQS or PTR methods. The LBR curve in this interval is thus primarily derived only from the BDC data.

### Origin of modern flora

**Figure 9:**
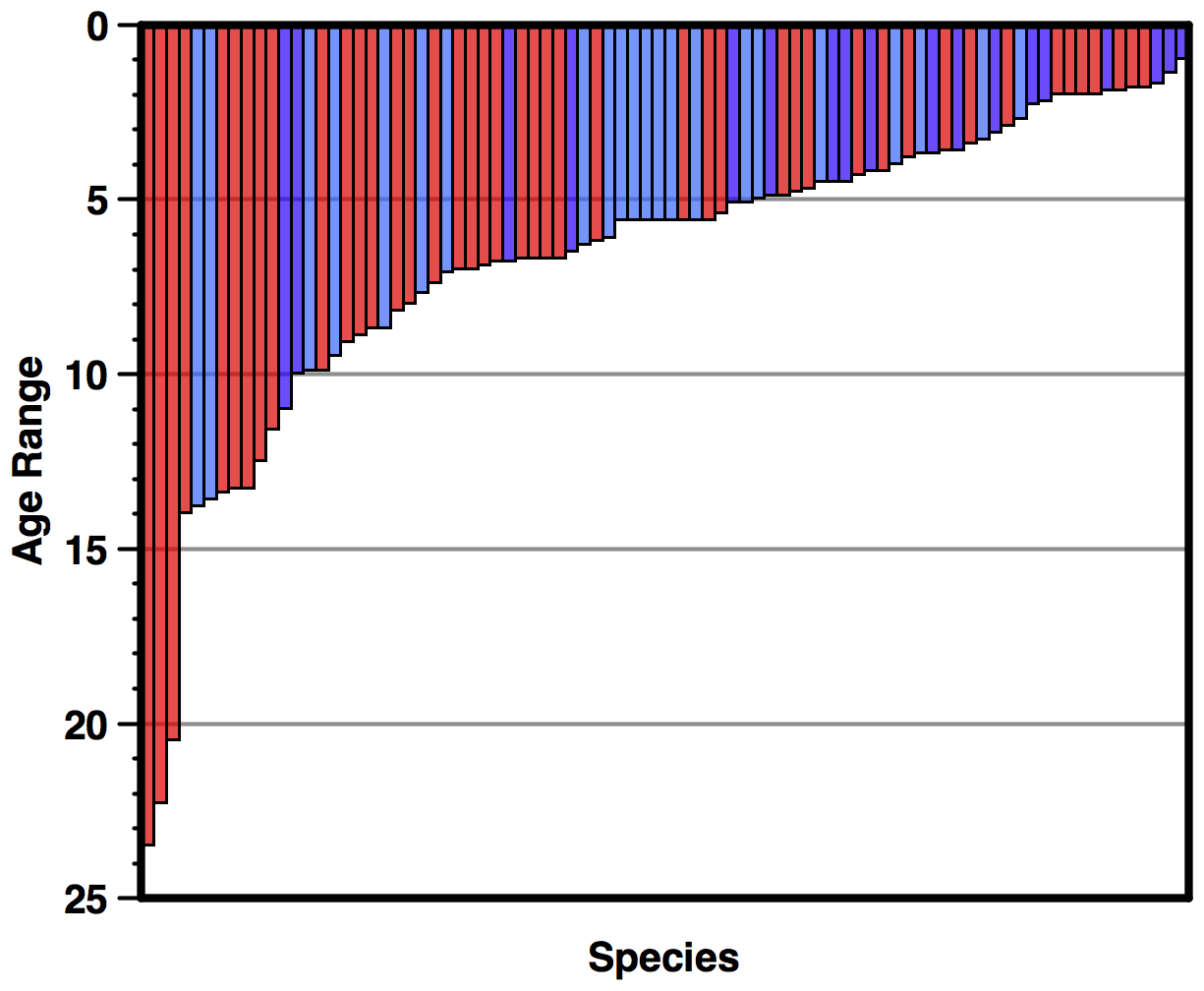
Origin of modern diatom flora, from stratigraphic ranges in the BDC. red - tropical; light blue - Southern Ocean; purple - North Pacific. Age scale: [42].

**Figure 10:**
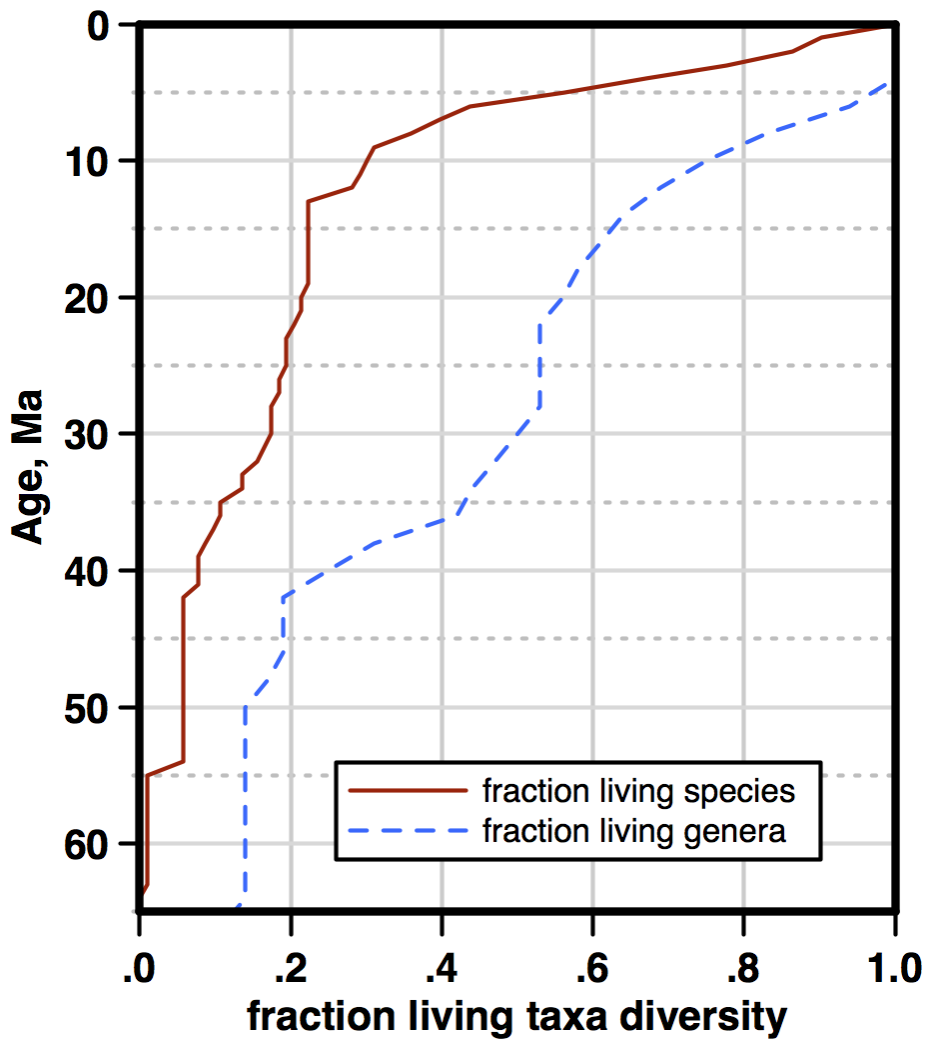
Fraction of living diversity in the BDC catalog present in older time intervals. Solid red line - fraction living species; dashed blue line - fraction living genera. Age scale: [42].

The first occurrences of all living species with origins in the Neogene from the BDC are shown in a range plot in Fig. 9, colored by biogeography. A shift towards higher origination rates for living species, and an increase in the contribution of polar taxa can be seen in the latest Miocene (ca 7-5 Ma) immediately preceding the early Pliocene warm interval (ca 4.5-3.5 Ma). Nearly 50% of living species originated <5 Ma; ca. 80% (and 50% of genera) in the Neogene (Figs. 9, 10). This suggests that Neogene, particularly late Miocene-Recent, diversity-environment relationships are most important in estimating future responses to climate change. Also, the recent flora is dominated by species originating in very cold latest Cenozoic conditions, and many of these are from the coldest (polar) regions (Figs. 8, 9).

### Diatom diversity and Cenozoic climate change

#### Diatom diversity and Cenozoic benthic oxygen isotopes

**Figure 11:**
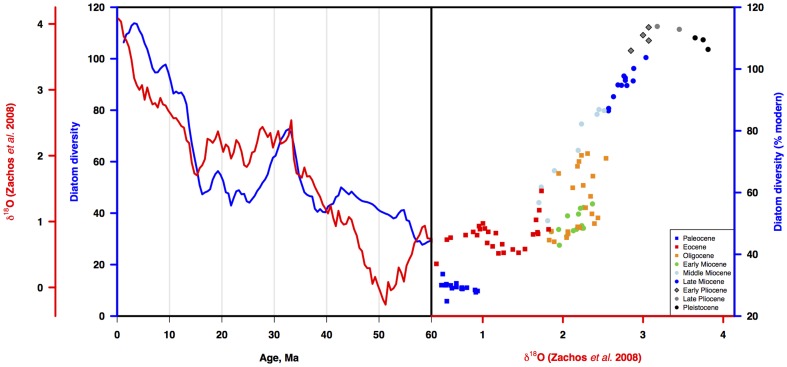
Diatom diversity and global climate proxy mean benthic ∂^18^O. a) Three point moving average of three diatom diversity time series estimates from figure 7 (blue line) vs time series of ∂^18^O [22] (red line). b) Diatom diversity vs ∂^18^O over the Cenozoic. Squares - Paleogene; circles - Neogene. Early Pliocene - diamond. Age scale: [43].

The diversity curve is strongly visually correlated to climate (Fig. 11a), with both the primary trend and many secondary fluctuations being similar between the two curves. A direct comparison (Fig. 11b) reveals the strong, but changing relationship between climate and diatom diversity. Paleocene-Eocene diversity is largely insensitive to climate, Oligocene-Miocene diversity is highly sensitive, while Pliocene-Recent diversity appears largely insensitive to climate, with the early Pliocene being a transition between very low and high sensitivity regimes. The correlation is statistically highly significant for the entire curve (r = 0.82, rho = −.88, p<.001), and even more so for the Miocene interval of rapid change (15-5 Ma, r = .98, p<.001).

#### Cenozoic carbon cycle (benthic carbon isotopes, pCO_2_) and diatom diversity

**Figure 12:**
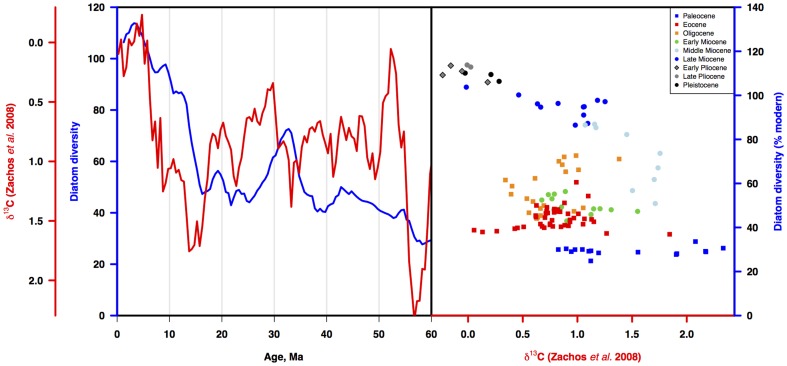
Diatom diversity vs carbon cycle proxy mean global benthic ∂^13^C. a) Three point moving average of three diatom diversity time series estimates from figure 7 (blue line) vs time series of ∂^13^C [22] (red line). Diatom diversity vs ∂^13^C over the Cenozoic. Symbols as in figure 11. Age scale: [43].

**Figure 13:**
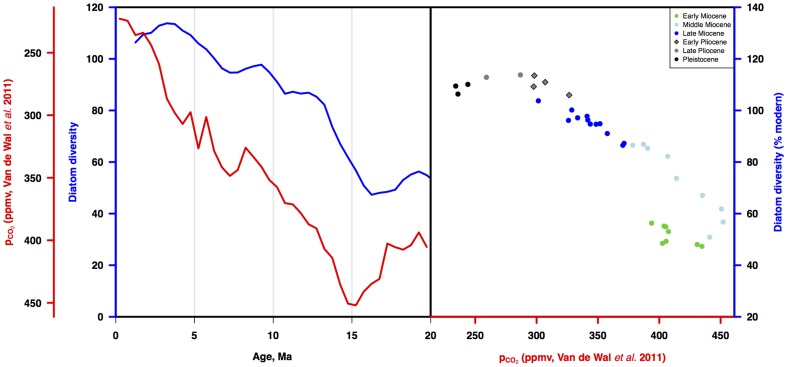
Diatom diversity vs estimated atmospheric pCO_2_ for the last 20 Ma. a) Three point moving average of three diatom diversity time series estimates from figure 7 (blue line) vs time series of estimated pCO_2_ [31] (red line). b) Diatom diversity vs pCO2. Symbols as in figure 11. Age scale: [43].

Comparison of the LBR diversity curve to the Cenozoic record of ∂^13^C (Fig. 12) shows that over most of the Cenozoic, diatom diversity and ∂^13^C were not correlated. Paleocene and Eocene variability in carbon isotopes was significant but not matched by equivalent variation in diatom diversity; more variable diversity in the Oligocene and early Miocene did not match shifts in carbon isotopes. Mid Miocene-Recent diversity (from ca 15-0 Ma) however is strongly correlated to the ∂^13^C record (r = .92, p<.001). Diatom diversity is also strongly correlated to the pCO_2_ record over the last 15 my (r = .92, p<.001; [31]; Fig. 13).

#### Robustness of correlation between diversity and geochemical parameters

Detrended analyses and analyses using alternate datasets (individual diversity estimates vs averaged; full Cenozoic vs only the 40-0 or 15-5/0 Ma time intervals) results are summarized along with undetrended analyses in Table 1. These show that the correlation between diatom diversity and climate is highly significant even in detrended data, or using variations of calculated diversity. Correlations are less for the full Cenozoic data but all significant (p<.05), and stronger for both the 40-0 Ma interval where the diversity estimates are most robust, and for the Miocene-early Pliocene interval during which most living species originated (p values all <.001). Various other tests (not listed), such as not excluding time bins with less than three diversity estimates in calculating average bin diversities adds data values primarily to the Paleogene, and has no significant effect on the observed degree of correlation, nor are there (with one exception - PTR in the Miocene-early Pliocene) significant differences in the p values for individual or combined diversity estimates.

**Table 1:** Correlations between diatom diversity and selected parameters.

	Pearson r	p value	Significance
LBR Diversity:			
Diversity/∂^18^O	0.818	<2.2E-16	***
Diversity/∂^18^O detrended	0.198	2.65E-02	*
Diversity/∂^18^O 5-pt average	0.823	<2.2E-16	***
Diversity/∂^18^O 5-pt average detrended	0.199	2.52E-02	*
Diversity/∂^18^O 0-40 Ma detrended	0.423	1.14E-04	***
Diversity/∂^18^O 5-15 Ma	0.975	2.83E-13	***
Diversity/∂^18^O 5-15 Ma detrended	0.938	1.02E-09	***
Diversity/∂^13^C	−0.401	3.30E-06	***
Diversity/∂^13^C detrended	−0.293	8.52E-04	***
Diversity/∂^13^C 0-40 Ma detrended	−0.331	3.06E-03	**
Diversity/∂^13^C (15Ma onwards)	−0.922	3.26E-12	***
Diversity/∂^13^C (15Ma onwards) detrended	−0.649	6.41E-10	***
Diversity/∂^13^C 5-pt average	−0.440	2.53E-07	***
Diversity/∂^13^C 5-pt average detrended	−0.318	2.90E-04	***
Diversity/pCO_2_ (15Ma onwards)	−0.925	2.04E-12	***
Diversity/pCO_2_ (15Ma onwards) detrended	−0.633	2.96E-04	***
Diversity/No of NSB samples	0.839	<2.2E-16	***
Diversity/No of NSB samples detrended	0.687	<2.2E-16	***
BDR Diversity:			
Diversity/∂^18^O	0.848	<2.2E-16	***
Diversity/∂^18^O detrended	0.242	5.21E-03	**
Diversity/∂^13^C	−0.477	7.35E-09	***
Diversity/∂^13^C detrended	−0.332	9.94E-05	***
Diversity/∂^13^C (15Ma onwards)	−0.928	1.68E-13	***
Diversity/∂^13^C (15Ma onwards) detrended	−0.888	6.15E-11	***
Diversity/∂^13^C 5-pt average	−0.457	5.88E-08	***
Diversity/∂^13^C 5-pt average detrended	−0.326	1.76E-04	***
Diversity/pCO_2_ (15Ma onwards)	−0.868	5.41E-10	***
Diversity/pCO_2_ (15Ma onwards) detrended	−0.519	3.30E-03	**

p<0.001.

0.001<p<0.01.

0.01<p<0.05.

Table S3 contains the main results of our analyses for use in further studies.

## Discussion

### Methodology

We argue that we have been able to extract a coherent diversity signal from incomplete, biased occurrence data, as demonstrated by the similarity between diversity estimates made using different methods, and using very different types of source data (raw occurrence data or range estimates from a catalog). An essential component of our reconstruction method from occurrence data is the use not only of SQS subsampling to address changing geographic structure in the occurrence data, but also the application of an evenness correction factor based on modeling. This latter correction is not normally used in paleodiversity studies, but in our data is shown to be very significant and necessary in order to obtain a coherent diversity signal. In a recent study of similar deep-sea microfossil data, Renaudie and Lazarus [36] implicated changing evenness in occurrence data as the primary reason why standard subsampling methods, including SQS, failed to recover an unbiased diversity history. Our modeling method is straightforward and may offer an effective solution for this problem, improving the accuracy of diversity reconstructions using occurrence data.

### Systematic biases in data

Despite obtaining a coherent diversity signal, our results may still be inaccurate if there are systematic biases in the data which would produce coherent biases in our diversity estimates. We specifically consider changes in preservation over time which might bias our results. The height of the late Eocene diversity peak may in part be a preservation artifact: older Paleogene diatoms are often diagenetically altered [13], [14] to widespread cherts [47], while decreasing silicification in Oligocene and younger species [14], and more corrosive waters due to declining oceanic silica concentrations, particularly since the Eocene-Oligocene boundary [48] may have reduced preserved diversity in these younger sediments. We conclude that our estimate of substantial increase in Cenozoic diversity is conservative, and may even underestimate the true total relative diversity rise.

### Diversity and productivity in living systems

Our results have implications for several fields. Many of these implications depend not on diatom diversity itself but diatom export productivity. We distinguish two distinct types of relationship between diversity and export productivity: naturally occurring behavior that has developed over evolutionary time; and perturbed relationships due to rapid extirpation or extinction, in which evolution has not had time to operate.

The relationship between diversity and productivity in living natural systems is controversial, and scale dependent. However there are several reasons to believe, on the global scale and over longer (but not geologic) time-scales, that biotic diversity and productivity are in general, positively related, even for groups where local diversity-productivity relationships show low diversity with very high productivity [49]. This appears to be true also for diatoms. Although on short time scales (weeks to seasonal), or in local environments diatom export productivity is associated with blooms dominated by very few species, over broader scales diversity and productivity appear to be correlated. In the modern Atlantic ocean living diatom diversity is strongly linked to diatom abundance/export of carbon (diversity:log biomass correlation r = .864, from data in [50], Fig. S1); productivity is significantly correlated to diversity also in freshwater plankton [51]. These results are in accord with global models of marine plankton diversity [52] in which high diversity (despite a slight geographic offset due to the inclusion of other phytoplankton functional groups) is associated with regions of high diatom export as indicated by sedimentary opal deposition [15].

Perturbed systems - To our knowledge, no experimental studies of short-term (annual to decades-centuries) pelagic ecosystem responses to species loss exist. However, hundreds of experimental studies with a variety of other systems, including not only terrestrial but also aquatic environments, have shown that ecosystem services and diversity are significantly correlated, with diversity loss reducing productivity and nutrient cycling [53], [54].

### Comparison of new diversity result to prior estimates

Having an independently derived estimate (LBR) for Cenozoic diatom diversity history, which we argue is reliable, we can compare this to prior estimates of diversity: the CSC and RS curves.

The RS diversity estimate is based, as the LBR estimate, on subsampling of the same occurrence database. Between ca 40 and 20 Ma the two estimates are quite similar, with an initial rapid rise from 40 Ma to a peak at the Eocene-Oligocene boundary, a decline throughout most of the Oligocene, and a more modest increase in the early Miocene. The curves are very different over remainder of the Neogene. The LBR curve shows a dramatic, rapid rise beginning at the base of the mid Miocene, reaching diversity levels, at their peak in the early Pliocene, ca 50% higher than the E/O peak, while the RS curve shows only a very gradual increase in diversity throughout the mid Miocene-Recent, reaching values only ca 75% of the E/O peak diversity in the Recent. As we have shown, the differences are due to the failure of the assumptions underlying the RS analysis in the mid-Miocene to Recent, e.g. no major changes in biogeographic endemism, or changes in relative abundance structure in the sampled populations.

The CSC estimate is a range through estimate of raw NSB data. It is similar in overall shape to the LBR curve, though this varies with time interval. The Eocene and older parts of the curves differ substantially: compared to the LBR curve the CSC curve shows no major peak in the late Eocene vs diversity fall in the late Oligocene, and a much steeper rise in the mid Eocene from low diversity values of the Paleocene-early Eocene. However over the last ca 30 Ma both curves show a plateau in diversity between ca 30 and 20 Ma, a rapid rise beginning ca 18-15 Ma reaching a brief peak in diversity in the early Pliocene slightly more than twice the prior plateau values, and a slight decline into the later Pliocene-Recent. The two curves differ substantially however in estimates of absolute diversity. For much of the Cenozoic, and in particular the last 30 Ma, the CSC diversity values are as much as 50% greater than those in the LBR curve.

Higher absolute diversity values in the CSC vs the LBR curves are difficult to explain as a simple calculation artifact: the LBR curve derives its absolute values via calibration to the BDR catalog curve, which is in principle also a range through estimate of diversity, derived even in part from the same deep-sea occurrence data, even if using different literature sources and additional onshore section data. The absolute values for the LBR curve should be thus similar, or, given the use of additional sources, somewhat higher than the CSC curve; the results however are the opposite. We speculate that the CSC curve contains substantial amounts of erroneous diversity due to data outliers and incomplete identification of synonyms in the taxonomy. The diatom taxonomy used in the Neptune database was only provisional when first compiled, and recent revision (Iwai et al, in prep; [55], not yet incorporated into NSB) has identified many additional synonyms. These may have inflated diversity in the CSC calculation in comparison to the absolute estimates in the more completely revised BDR catalog. Data outliers, due to age model errors, reworking and mis-identifications, may individually contribute only a small amount to inflated diversity estimates but cumulatively, particularly in range through calculations, may have also increased diversity by significant amounts. The use of range through methods at least tends to distribute local data errors of the sort described above over several time bins, so the net result of such error might well be to inflate local absolute values but leave the overall relative shape of the curve largely unchanged.

Why should however the relative pattern of diversity change be so similar between the LBR and CSC curves? One possibility is that the CSC curve is derived from a completely sampled record, so that changes in sampling had no effect on the diversity estimate. This is not in accord with the strong overall and detrended correlation between raw NSB diversity and number of samples (Fig. 5). Nor is this in accordance with our simulations of diversity vs sample size (Fig. 3) - 90% sampling completeness of diversity for a simulated diversity of 100 is only reached in the Neogene diatom data with sample sizes of ca 10,000 - values of data density not even approximated in the NSB data except for a few bins >5,000 but <7,000 in the Plio-Pleistocene. At more typical Neogene sample densities/bin of ca 1,000 (Fig. 5a), only ca 2/3 of the true diversity is recovered (Fig. 3a), and there is a significant slope showing that diversity does vary with sample size.

**Figure 14:**
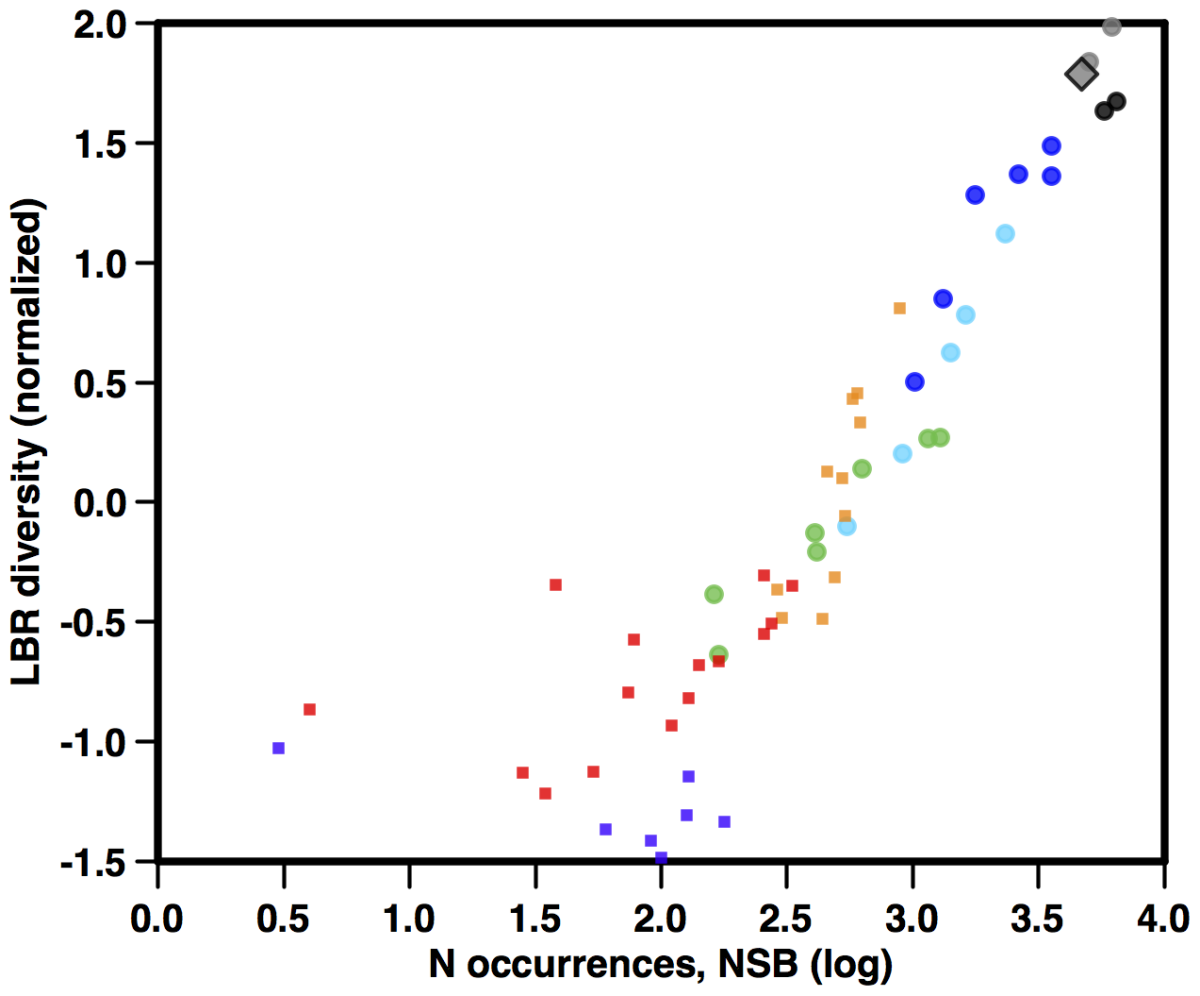
Diatom occurrences per time interval in the NSB database (log scale) vs LBR consensus diatom diversity estimate from figure 7. Symbols as in figure 11. Age scale: [43].

Given the very strong correlation of CSC diversity and sample size at all intervals over the Cenozoic, the correlation seen between our best estimate of diversity (LBR) and CSC curves over the last 30 Ma can only be due to a strong correlation between LBR diversity and sample availability over the last 30 Ma. This is shown in Fig. 14, where a very strong correlation can be seen for this time interval (r = .84, p<.001), in contrast to the Paleocene-Eocene, where no correlation is apparent. Why should this be, since the LBR diversity has been computed to be independent of sample size? Although the first order trend may well be coincidence (due to both series increasing rapidly towards the Recent), the correlation of detrended series (Fig. S2) is significant (r = .69, p<.001) and requires explanation. We suggest that diatom diversity may have influenced sample availability, thereby to some extent inverting the assumed relationship between variables. This is because diatom diversity has likely been causally correlated to the relative abundance of siliceous sediments in each sampled interval (Renaudie and Lazarus, in prep; see also ‘Diversity and Climate’ below). Unlike most fossils, where abundances in fossil-bearing sediments depend primarily on external geologic factors such as sea-level that regulate creation of sediment and its preservation after deposition, deep-sea sediments that have reasonably well-preserved fossil diatoms (diatom oozes and admixtures of siliceous ooze with carbonate and clay) are to a large extent created by the abundances of diatoms themselves in the overlying water column ([56]). The relationship to diversity can either be a direct influence of diversity on the relative abundance of diatoms in the plankton (the effect of more diverse evolutionary adaptations on relative nutrient capture), or indirect via the response of both diatom abundance and diatom diversity to changing amounts of nutrient input into the oceans due to changing Cenozoic climate. These hypotheses are not mutually exclusive and a mixture of direct and indirect response is feasible. Testing these ideas are however beyond the scope of our current study.

### Diatom diversity and evolution of other groups of organisms

We group our discussion of implications into those related to the diversity history and its correlation to the evolution of other groups of organisms; and those related to the correlation between diversity and climate. In the first category we cite three examples.

#### Grasslands

Rabosky and Sorhannus [18] suggested that the relatively low post-Eocene diversity of diatoms in their reconstruction argued against both a grassland expansion-driven increase of silica to the oceans, with consequent diversification of marine diatoms; and against co-evolutionary, inverse relationships between Cenozoic diatom and coccolithophore diversification. Our results do not support their arguments, although there are other reasons to question a grassland-marine diatom hypothesis, as proposed by Falkowski et al. [7]. Because grasses can alter the extent to which weathering products are stored in soils prior to their being dissolved and transported by water, it is reasonable to propose relatively short-term, local effects of different vegetation on local freshwater bodies [57]. It is not clear however if this scales to the global oceanic silica cycle over millions of years. Regardless of the silica content of standing grassland biomass, significant effects on the marine silica budget can only come from long-term (>10^3^ year) differences in net rates of silicate weathering, yet grasslands may not actually increase long-term weathering rates vs. other vegetation types [32]. Grasses also were evolutionarily well developed before their late Neogene ecologic expansion [58]. Grassland evolution and ecologic expansion may thus not have driven diversification of marine diatoms. It is more likely both were influenced by tectonically driven Neogene increases in silicate weathering surfaces with consequent increase in marine nutrients, CO2 drawdown, cooling of continental climate and cooling climate forcing of enhanced ocean circulation [32], [59], [60].

#### Cetaceans

Our results weaken support for the hypothesis that Cenozoic cooling and the consequent concentration of high export productivity in upwelling regions, dominated by efficient, short food-chain diatoms, provided high levels of large zooplankton/nekton food supply that supported the radiation of cetaceans [60], [61]. Although the Cenozoic-scale pattern seems plausible [60], and multiple factor models may still be valid [61], we did not, as in [61], find a significant correlation between latest Oligocene-Recent Cetacean and diatom diversity (maximum r values of ca. 0.2, p>0.1: Fig. S3 and Table S2). We note however that the number of data points is small, and our method of averaging different curves means that, although the overall data series is robust, the precise values of a small number of points is not likely to be constant in alternate computations. Neither our negative, or the earlier [61] study's positive results should thus be considered a strong test of the hypothesis.

#### Radiolarians

Lazarus et al. [48] proposed that expanded Cenozoic diatom export productivity, together with increasing water column stratification, led to reduced silica availability in low latitude waters and an evolutionary reduction in radiolarian silica use, but did not statistically test diatom-radiolarian correlations. Our results support their inferred influence on radiolarians of an increase in diatom productivity over the Cenozoic, with a strong negative correlation of Cenozoic radiolarian silicification to diatom diversity (r = .86, p<.001; Fig. S4), although, as silicification data are few and noisy, detrended tests are not significant (p>.1).

### Diversity and Climate

#### Environmental context of Cenozoic diatom evolution

As we wish to consider how the diversity history of diatoms is correlated to changing environments it is useful to review the most relevant aspects of Cenozoic ocean change. The main features are well known [62] and need be only briefly mentioned here. Early Cenozoic oceans were relatively warm, with little biogeographic differentiation. After reaching maximum warmth in the early/mid Eocene, ocean surface temperatures began to cool, and polar regions and tropical regions began to be more strongly differentiated from each other in the late Eocene. Cold deep-water circulation between the poles, underlying a more strongly isolated warm low to mid latitude surface water region developed along with Antarctic glaciation at the Eocene-Oligocene boundary. The late Oligocene to mid-Miocene were marked by a general warming trend, although punctuated by brief cooling episodes such as the M1 glacial event at the Oligocene-Miocene boundary. Renewed cooling near the end of the mid Miocene and associated expansion of the Antarctic ice-sheets was associated with the development of mid-latitude coastal upwelling systems. Cooling was briefly interrupted by a relatively modest warming interval in the early Pliocene, before being resumed in the late Pliocene-Recent, in association with widespread northern hemisphere glaciation. In parallel to this ocean climate history, both atmospheric pCO_2_ and rates of weathering (which provide nutrients for new diatom productivity) were changing. Although still very poorly constrained, with different proxies often yielding very different estimates, Cenozoic pCO_2_ showed a largely parallel trend, from low initial values in the Paleocene-early Eocene, maximum values of ca 1000 ppm in the mid-late Eocene, and declining values in the Oligocene-Recent (with modern pre-industrial pCO_2_ values of ca 280 ppm) [63]. Weathering rates are even more poorly constrained, and, when not computed directly from pCO_2_, are argued, based on various geochemical proxies, to either have increased substantially in the Oligocene-Recent [64] or have remained, at least for the Late Miocene-Recent, relatively constant [65].

Cenozoic climatic-ocean history was long believed to have been primarily driven by the tectonic opening of gateways that altered ocean circulation and polar heat transport, such as the development of a circumpolar Antarctic current near the Eocene-Oligocene boundary [56], but more recently changing concentrations of atmospheric pCO_2_ have been implicated as the primary driver of Cenozoic climate change [66]. The role of ocean gateways however was not negligible, and this affects in particular our ability to interpret the causes of late Miocene to Recent changes in diatom diversity. Our understanding of Late Miocene and early Pliocene oceans is still very incomplete and rapidly evolving, and current understanding is thus worth summarizing.

Late Miocene climate was probably only moderately warmer than the Present (2–4°C globally: [67], however with several degrees additional warming in high latitudes: [67], [68]), and thus not much warmer than the early Pliocene [69], [70]. The early Pliocene is specifically noted as it is often used as a past proxy for projections of future climate warming [69], although the pCO_2_ concentrations of the early Pliocene may not be representative of more extensive future warming, particularly beyond 2100 [66], [71]. Late Miocene-early Pliocene climate was driven by an as yet not fully resolved combination of pCO_2_ (via atmospheric temperature and wind fields) and unique, non-recurring past events such as the tectonically driven partial or complete closure of several oceanic gateways. The Panama gateway in particular closed gradually in the late Miocene-early Pliocene, which redirected tropical Atlantic waters northwards, warming the North Atlantic, and increasing pole-to-pole (Norwegian-Greenland Sea to Southern Ocean) surface-to-deep circulation and Southern Ocean diatom productivity [60], [72], [73]. Paleoceanographic studies suggest that the initial partial closure of the Panama gateway had already established the modern pattern of circulation by the early late Miocene [72]–[74], although final closure did not occur until the earliest Pliocene. Biogeographic, abundance frequency and evolutionary rate data for siliceous plankton [36], [75], [76] also suggest that an essentially modern global biogeographic pattern was already established by the early late Miocene. Moderately warmer than present late Miocene ocean climates, despite very low late Miocene pCO_2_ estimates from epsilon-p plankton alkenones (ca 250+/− 50 ppm - equal to or even below modern pre-industrial values) [68], also have suggested that these past tectonic controlled circulation patterns rather than pCO_2_ were primarily responsible for late Miocene ocean conditions.

More recent work however calls this tectonic ‘gateway’ model of late Miocene oceanography into question. Several ocean-atmosphere models have found Cenozoic gateways to have had (compared to pCO_2_ or non-tectonic paleoceanographic mechanisms) only a secondary impact on global climate [68], [77]–[80], while new studies suggest higher, if still moderate (ca 250–450 ppm) late Miocene pCO_2_ [31], [78], [81]. It is important also to note that, while modeling studies provide valuable insight into the relative importance of causal factors in climate change, their ability to reconstruct absolute values of past climate is still limited. In particular, climate models tend to systematically and substantially underestimate the extent to which polar conditions warm, either as the result of changing pCO_2_ or due to changes in gateways or other boundary conditions [77], [82].

The late Miocene surface ocean conditions that these different controlling factors created are known to have differed from the modern, including for diatoms relevant environmental controls such as circulation, fronts, and distribution of nutrients. As documented by numerous deep-sea drilling sediment sections, biosiliceous sediments were more widespread in polar regions, indicating a reduced extent of permanent sea ice, even in regions near the Antarctic coast [83], [84], while in lower latitudes the surface-deep ocean density contrast was lower, but with a deeper thermocline [68], [85]. Surface water temperatures were also higher, although only by at most a few degrees [82]. Surface water temperature estimates from polar regions are however sparse, due to the general scarcity of carbonate-shelled plankton in high-latitude late Neogene pelagic sediments. How these conditions might relate to diatom evolution are discussed below.

#### Abiotic controls on large scale patterns of evolution

The relative importance of abiotic vis biologic interactions as shapers of large-scale patterns of evolution is a central question in evolutionary biology, but available data are few and difficult to interpret [86]. Most studies of plankton have so far shown a rather complex, episodic or threshold correlation of diversity to environment. This includes correlations to extreme events such as the abrupt mass extinction at the K/T boundary due to meteor impact, as well as many more minor climatic events (cooling, anoxia and others) in the Cretaceous and Cenozoic [38], [87], [88]. Only a few studies using general statistical comparisons of entire time series of diversity and environmental data have been done (e.g. Ezard [27] on calcareous zooplankton), and these have found significant if also complex and intermittent control by environmental factors, with single factor correlation coefficients of ca 0.4 in detrended data [27], similar to coefficients seen for Phanerozoic fossil invertebrates [89]. Our results for Cenozoic marine plankton diatom floras confirm an abiotic control of diversity, but differ from prior results in that: the correlation between diversity and climate is remarkably strong (r of ca 0.6 in detrended data; Spearman's rho >0.9 in the raw data), and unlike prior results e.g. [27], [89], in diatoms colder climate, not warmer, is correlated to higher diversity.

We speculate that the higher degree of correlation between diversity and climate in diatoms, vs calcareous zooplankton or marine invertebrate benthos, may reflect the more direct impact that the physical environment has on phytoplankton growth, via regulation of temperature, light and nutrients. Zooplankton or invertebrate benthos by contrast are higher in the food chain and nutrient-climate correlations are reduced. This idea however ignores other aspects of phytoplankton growth such as grazing by zooplankton, and other pathways by which climate can influence invertebrate and zooplankton diversity, such as temperature effects on the energetics of carbonate shell formation. Comparative studies (e.g. using similar methods) of other microfossil records with complementary ecologies and shell mineralogy (calcareous phytoplankton: coccolithophores; siliceous zooplankton: radiolaria) might provide more insight. Currently, the best available estimate for Cenozoic calcareous phytoplankton diversity [38] shows a largely dissimilar diversity history to Cenozoic diatoms. Despite sharing an interval of low diversity in the late Oligocene-early Miocene, the overall trend in calcareous phytoplankton diversity has been downwards over the Cenozoic, from a diversity maximum in the earliest Eocene, which is regarded as the warmest interval of the Cenozoic. The different trajectories of diatoms vs calcareous phytoplankton has generally been interpreted to reflect the groups’ contrasting ecologic adaptations to mesotrophic-oligotrophic (calcareous plankton) vs eutrophic (siliceous plankton) water conditions. These shifted from broadly mesotrophic conditions in the earlier Cenozoic towards regionally partitioned oligotrophy and eutrophy as cooling climate increased localized upwelling [7]. Regional upwelling specifically increased in the Southern Ocean after the Eocene-Oligocene boundary, in equatorial open ocean systems, and in mid-latitude coastal upwelling systems, such as the Benguela and California systems, in the late Miocene. The oceans thus came to be more strongly divided into a broad, geographically interconnected, low to mid latitude warm surface water, more strongly oligotrophic ocean dominated by calcareous phytoplankton, and a series of distinct, separated regional areas in both the tropics and high latitudes of more eutrophic waters dominated by differentiated endemic floras of siliceous plankton. This pattern has long been known [13], [14] but our results (e.g. Fig. 8) provide a quantitative evaluation of this phenomenaon, and show that diversification in Cenozoic diatoms was due primarily to the increased diversity resulting from geographic endemism between these distinct regions. Since the mid Eocene tropical diversity has increased from ca 35 species to a maximum in the Pliocene of 60 species (ca 25 species, <2× relative regional increase), while overall diversity, e.g. including endemic polar floras, increased from ca 55 to 145 species over the same time interval (ca 90 species, 3.6× the tropical species increase, >2.6× relative regional increase). There is thus a strong geographic cause in addition to the physiologic/ecologic cause (oligo- vs eutrophy) in Cenozoic diatom diversification. It can be argued that the primary reason for Cenozoic diatom diversification is not that diatoms are better adapted to increasing Cenozoic eutrophic environments, but that they are better adapted to Cenozoic environments that are geographically distinct.

#### Diatoms and Cenozoic geochemical cycles

Much of the earth-sciences interest in global Cenozoic diatom diversity lies in the presumed correlation of increasing Cenozoic diatom diversity to increasing Cenozoic export productivity by diatoms in the oceans, which in turn may have substantially affected the Cenozoic evolution of the global carbon cycle, including atmospheric pCO_2_. Until quite recently [26] no direct sedimentary proxy for global diatom export productivity has been available, but it is generally thought that biogenic opal in marine sediments (largely consisting of diatoms) is a good, if rough, approximation of high diatom export productivity in the overlying water column [90]. The relative abundance of siliceous deep-sea sediments (vs carbonate or terrigenous) over the Cenozoic should thus indicate, at least approximately, the relative importance of diatoms to global oceanic export production. Our first order trend in diatom diversity is of major increase over the Cenozoic, suggesting a similar large increase in diatom export productivity and the relative abundance of biogenic opal in sediments. Prior compilations of biogenic opal abundance in Cenozoic deep-sea sediments [23]–[25] suggest that siliceous sediments have indeed become more common over the Cenozoic, although as these compilations were of a qualitative nature no numeric comparison is possible. Studies over shorter time intervals exist but are often difficult to evaluate. Cortese et al. [91] quantitatively studied details of the shifting geographic patterns of opal deposition in the late Neogene, but did not estimate opal relative abundance on a global basis, making comparison to our results difficult. Although no relative abundance estimates of siliceous sediment were given, recent studies [47], [92] calculated absolute Cenozoic diatom sediment abundance histories from, respectively, the MRC and Neptune databases. These estimates are likely to be affected by biases in the relative amounts of siliceous vs non-siliceous data entered per time interval by the database compiler (in both instances overseen by the senior author of this paper). Lastly, although only a regional estimate, dissolved ocean silica usage, estimated from silicon isotope gradients in the water column, increased in the Southern Ocean in the late Eocene between ca 37–35 Ma, which is interpreted to reflect increasing diatom export productivity [26]. This matches well our calculated increase in both global and Southern Ocean diatom diversity at this time. Thus, available data on changing Cenozoic opal export is very limited, but tends to parallel the diversity increase in diatom diversity seen in our study, supporting, albeit only weakly, the use of diversity as a proxy for opal export productivity.

While export productivity is important to understanding many aspects of the marine ecosystem, of particular interest is the effect that marine export may have had on the global carbon cycle, and thus on atmospheric pCO_2_ and climate. As noted earlier, ∂^13^C is often used as a proxy for global carbon cycle behavior. Late Neogene ∂^13^C shows a substantial shift (ca 1 per mil), which has been interpreted as reflecting an increase in the fraction of organic carbon sequestered by less ^13^C depleted groups of organisms, specifically C4 terrestrial plants and marine diatoms [30]. Our diatom diversity curve shows a close correlation to this carbon isotopic shift beginning in the mid Miocene. More generally, diatom diversity changes (in detrended time series) are positively correlated with coeval changes in detrended ∂^13^C since the early Oligocene. These comparisons thus provide qualitative support for the idea that increasing Cenozoic diatom diversity is correlated to increasing fractions of diatom export productivity, and increasing relative abundance of diatom opal in sediment, since the mid Miocene. Together with evidence from living ecosystems and the limited independent evidence for Cenozoic opal productivity (reviewed above) our results suggest that diatom diversity can be used to some degree as a proxy for the influence of diatoms on marine export productivity and the carbon cycle. Diatom diversity is further strongly correlated to pCO_2_ over the last 15 my. Since the pCO_2_ record is derived from the ∂^18^O record [31], this is not too surprising, but it is interesting that the correlation between diversity and pCO_2_ is stronger than between diversity and ∂^18^O, implying that the observed correlation to pCO_2_ is not simply one inherited from the observed correlation of diversity and ∂^18^O. Our analysis thus also suggests that increasing diversity and abundance of diatoms are closely linked to late Neogene changes in the global carbon cycle. Whether this latter link is due to both diatom diversity and pCO_2_ being driven by a third factor, such as increasing late Neogene silicate weathering, is however beyond the scope of our current study (see also below).

### Implications of correlations for response to future anthropogenic global warming

Our analysis shows that warmer oceans are associated with lower diatom diversity, suggesting the possibility that future warmer oceans due to anthropogenic warming may result in lower diatom diversity, i.e. extinctions, with possibly substantial consequences for the functioning of the ocean carbon pump. This is suggested both by the correlation of increasing diversity with increasingly cold climate in the Neogene, and the reduction of diversity that occurred with warming ocean conditions in the late Oligocene. The broad uniformity of response over such a long range of time, and under such a wide variety of ocean conditions (as reviewed above) suggest that response will be similar also in future climate change. There are however significant problems in converting these general correlations into concrete predictions of diatom response to future warming. Oligocene floras were, as we document, almost entirely composed of species not present today, and even many genera were different. It is thus difficult to be sure that the response behavior of the modern, taxonomically different flora would be the same. The diatom floras of the Late Miocene ocean were much more similar taxonomically to those of the present, and the diatom diversity-climate relationship is particularly sensitive in this time interval. Diatom diversity in the late Miocene was, in our estimate, up to ca. 20% lower than modern values (and up to 50% lower in the middle Miocene, ca 15 Ma), and important cold-water polar species, such as F. kerguelensis and Neodenticula seminae, which play a major role in export productivity in the modern ocean [5] were absent. Given the potential importance of this issue, and the lack of any prior estimate of marine diatom extinction risk, it is important to consider this question. Evaluating the probability of future extinction however requires evaluating a chain of cause-effect processes that links past pCO_2_->global climate->marine ocean conditions->diatom diversity->the carbon pump.

Although still uncertain, current paleoceanographic studies suggest that late Miocene ocean conditions might be a relevant analog for future marine plankton diatoms due to global climate warming, particularly on longer (post 2100) time scales. Reconstructions indicate higher temperatures and reduced sea-ice extent in polar regions, factors that are known to play a major role in the distribution of living diatom species and other phytoplankton, and diatom export productivity [50], [93], [94]. It thus seems reasonable to presume these factors, which, from our results are temporally correlated with lower diatom diversity, might also affect future diatom diversity, even if the proximal mechanisms are largely still unknown. Unlike the late Miocene however, where diversity-productivity relationships were in at least approximate evolutionary equilibrium, future climate change is expected to be orders of magnitude more rapid, so that rapid species loss would not be compensated for by significant evolutionary response, e.g. the result would be a perturbed system.

The effect of loss of diatom diversity on future ocean productivity is unknown but potentially significant. Bopp et al. [2] model a substantial drop in diatom abundance, particularly in high latitudes, in a 4X pCO_2_ climate change scenario, although only a moderate impact on the ocean biologic pump. In Bopp et al. [95], this moderate degree of global change in pump efficiency is in part due to increased pump activity in polar regions, which partially compensates for an a broader drop in pump functioning in lower latitudes. These models however assume future diatom plankton response, particularly in polar regions, can be extrapolated from the living plankton flora. Would this still be true if important diatom species, particularly in polar regions, were to become extinct? Would extinct species of diatoms be replaced by fully functionally equivalent (e.g. for export productivity) other diatom species, by functionally less efficient taxa, or functionally very different coccolithophores or non-skeletal plankton? Our results, together with evidence that rapid diversity loss is linked to reduced productivity [53], [54], suggest that loss of diatom diversity in future oceans, if it occurred, would indeed affect diatom abundance behavior, diatom-carbon export and thus further modify the global ocean pump, with consequences for future ocean regulation of pCO_2_.

While the above presents arguments why the correlation of diatom diversity to climate state should be considered in thinking about future climate change, there are limits as well. We argue that while our results provide a useful perspective on eventual system response, a variety of issues, including temporal scaling and other potential casual factors, limit our ability to use our results to make any direct estimate of extinction risk due to anthropogenic change. As reviewed above, Late Miocene oceans, although largely very similar to the modern ocean, were still dissimilar in details of circulation and climatic context. The proximate conditions that affect diatom distributions - nutrient concentrations, water column stability, frontal structures; and the variability of these on seasonal to multi-decadal scales, could have been significantly different: not only from those of the modern ocean, but also to the oceans that anthropogenic global warming will produce. Our understanding of these proximate controls in Late Miocene oceans is very sketchy, particularly in high latitude regions which are most important to understanding diversity and productivity response. Also important is the temporal scaling between our data and future global change in how global nutrient are input to the system, which at the time scales of our study may exert a rate limiting effect on climate change, since the residence times of key elements in the oceans are much less than the scale of our analyses (Silica ca 20 kyr; Carbon ca 200 kyr: 30,90). Although the proximate controls on past diatom diversity might have been temperature or other rapidly acting factor, if nutrients were the limiting factor, changes in weathering may well only affect oceans over time scales of many hundreds or thousands of years. It is thus difficult to use only a generic proxy such as ∂^18^O to identify truly analogous conditions in the past that can be used to predict future diatom behavior in a warmer world. In order to do this, we need much more information on the proximate controls on individual species distributions, both for now extinct species, and for modern species, whose ecology also is often only poorly known.

## Conclusions

Cenozoic diatom diversity and its correlation to climate change is important to understanding how evolution in pelagic systems functions, and how biotic diversity may regulate climate change itself over various time scales - from millions of years, to those expected for future anthropogenic global warming. Existing estimates of diatom diversification have been contradictory and have methodologic problems. We derive a new, internally coherent estimate using multiple methodologies and datasets, using a newly created, first-ever comprehensive catalog of Cenozoic marine diatom species ranges as well as a new version of the Neptune marine microfossil database, while explicitly controlling for prior problems in diversity reconstruction such as changing sample sizes, evenness of population frequencies - by use of a novel modeling-based correction function, and other factors. We show that diatoms diversified strongly over the Cenozoic, with major increases near the Eocene/Oligocene boundary, and in the mid Miocene. Diversification occurs primarily by increasing diversity of endemic high latitude floras, and the disjunct development of high productivity regions in Cenozoic oceans is suggested to be a primary reason for diversification of the group. Diatom diversity is strongly correlated to evolutionary characteristics of other groups of organisms such as radiolaria; and to the oxygen isotope proxy record of global climate change: diversity not only increases with intervals of cooling, but also significantly decreases during intervals of past warming such as the late Oligocene-early Miocene. Over the last 15 my diatom diversity is strongly correlated to both the global carbon isotope record and to estimated past atmospheric pCO_2_. These correlations suggest that diatoms have played an important role in the evolution of mid-Miocene to Recent climate, via their prominent role in the oceanic carbon pump. The correlation of warmer climate to lower diversity also suggests that global warming could potentially place a significant fraction of diatom diversity at risk of extinction, particularly as we show that important export productivity species originated only in the last few million years in association with the development of cold polar oceans. Both the time resolution of our study (0.5–1.0 my resolution) and the complexity of cause-effect relationships however mean that we cannot evaluate from our data alone the likelihood of future extinction over the next decades or centuries as a possible consequence of anthropogenic global warming.

## Supporting Information

Figure S1 **Number of diatom species vs biomass in water column samples from Atlantic ocean transect, from data presented in** [**50**]. Age scale: [43] **.**
(TIFF)

Figure S2 **Detrended numbers of occurrences in time intervals in the NSB database vs detrended LBR diatom diversity estimate.** Data point symbols age coded according to main paper figure 11. Age scale: [43].
(TIFF)

Figure S3 **Comparison of species diversity in Neogene cetaceans (red: sampled in bin and range-through) to diatom diversity of this study (blue: 3-point moving average of raw data and Neogene portion of Cenozoic detrended values as reported in table ST2).** Cetacean data from [61]. Age scale: [42].
(TIFF)

Figure S4 **Diatom diversity (average of the three estimates for a bin, not smoothed) vs radiolarian shell silicification, from** [**48**] **.** Silicification data binned to 1 my intervals, intervals with no silicification data excluded. Age scale: [43].
(TIFF)

Table S1 **The BDC (Barron Diatom Catalog).** a) Data. b) List of publications/sources used.
(TXT)

Table S2 **Pearson correlation coefficient r and color coded p value intervals for diatom diversity, Neoceti diversity (from** [**61**] **) and geologic age.**
(TIFF)

Table S3 **Main data and results of this study in numeric form.**
(TXT)
