# Supplementary figures and images for: Cenozoic Planktonic Marine Diatom Diversity and Correlation to Climate Change
Source: PLoS One. 2014 Jan 22;9(1):e84857. doi: 10.1371/journal.pone.0084857 (PMC3898954; doi:10.1371/journal.pone.0084857)

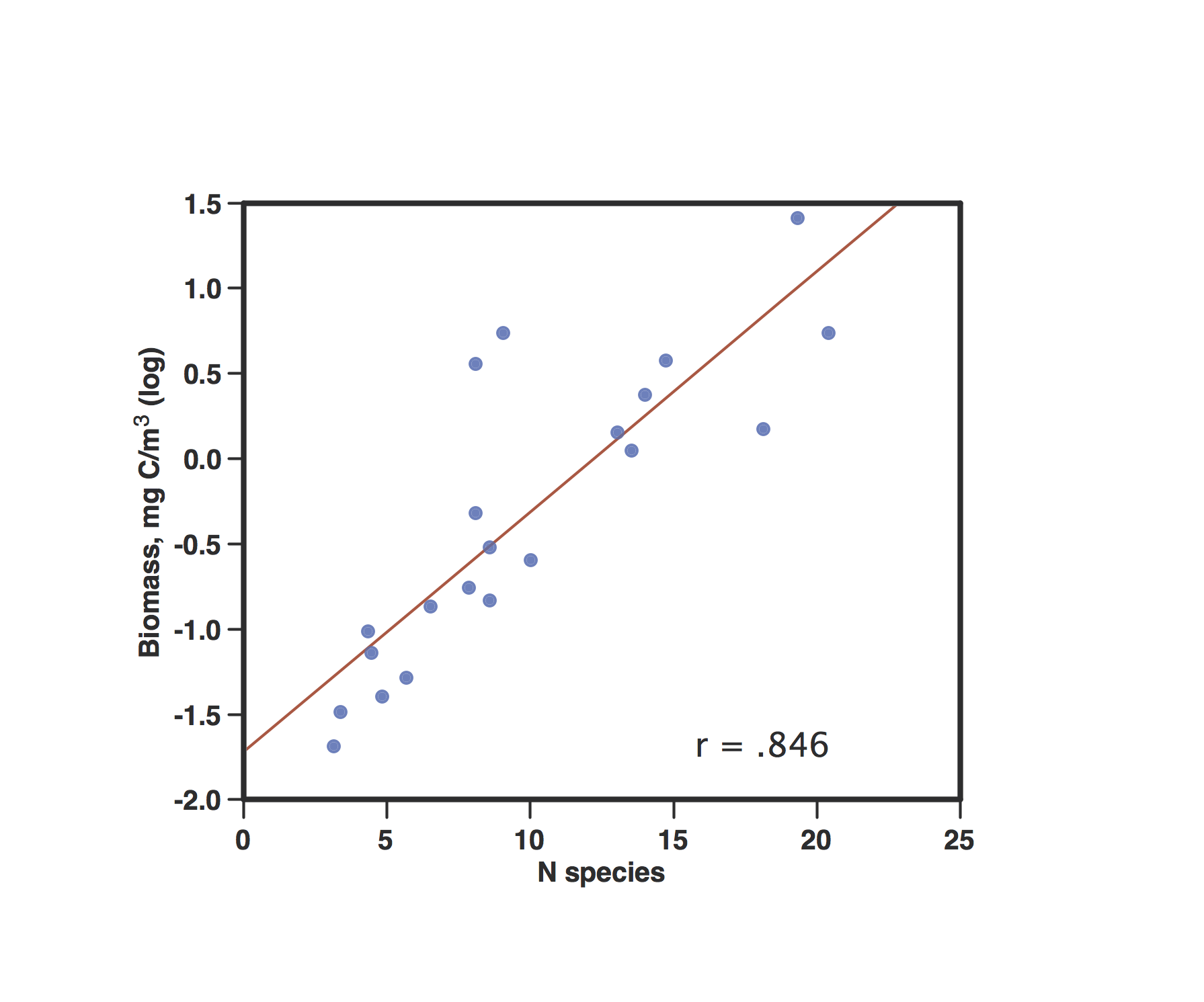

Supplement: Figure S1 — Number of diatom species vs biomass in water column samples from Atlantic ocean transect, from data presented in [50]. Age scale: [43] . (TIFF) [file pone.0084857.s001.tif]

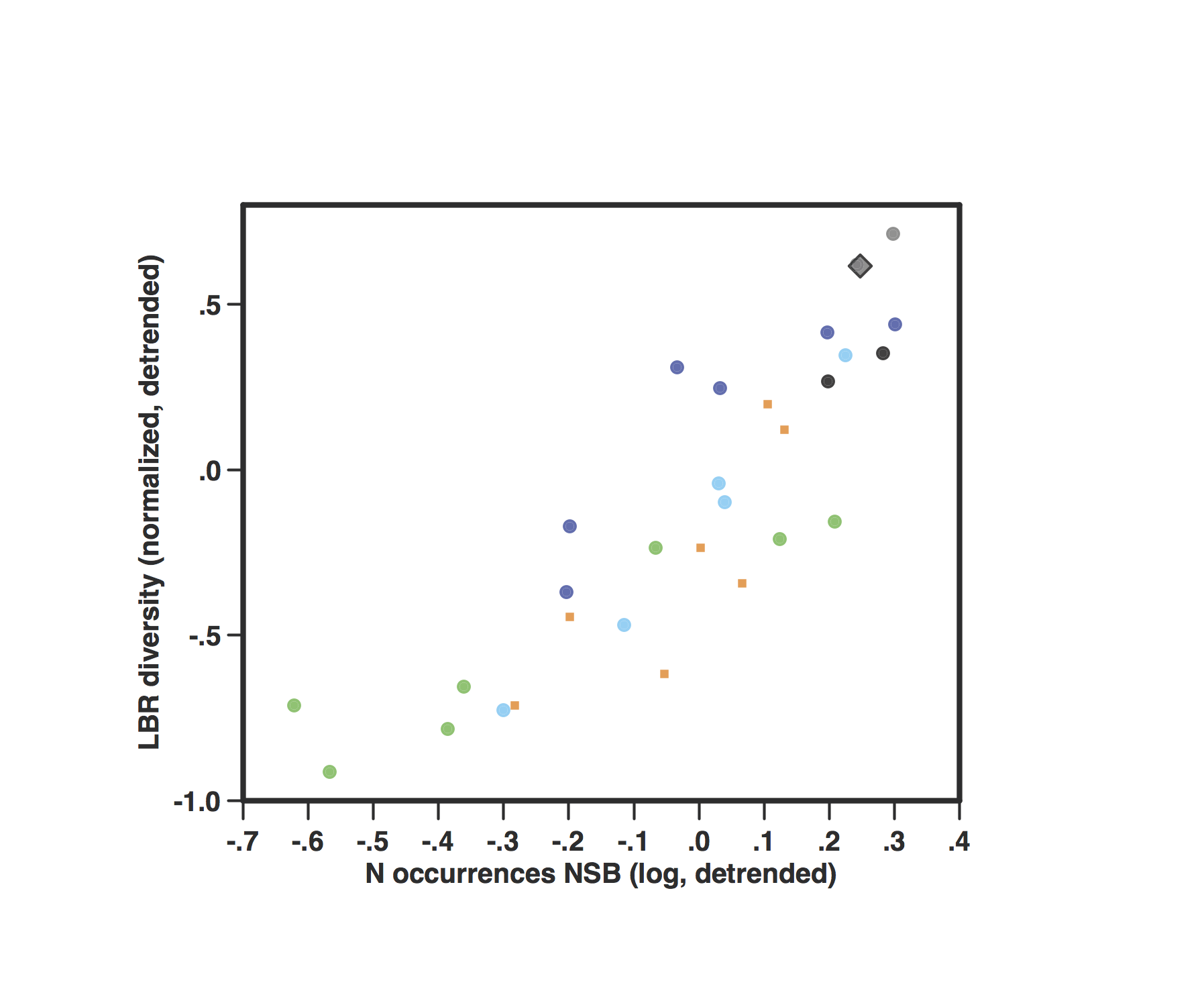

Supplement: Figure S2 — Detrended numbers of occurrences in time intervals in the NSB database vs detrended LBR diatom diversity estimate. Data point symbols age coded according to main paper figure 11. Age scale: [43]. (TIFF) [file pone.0084857.s002.tif]

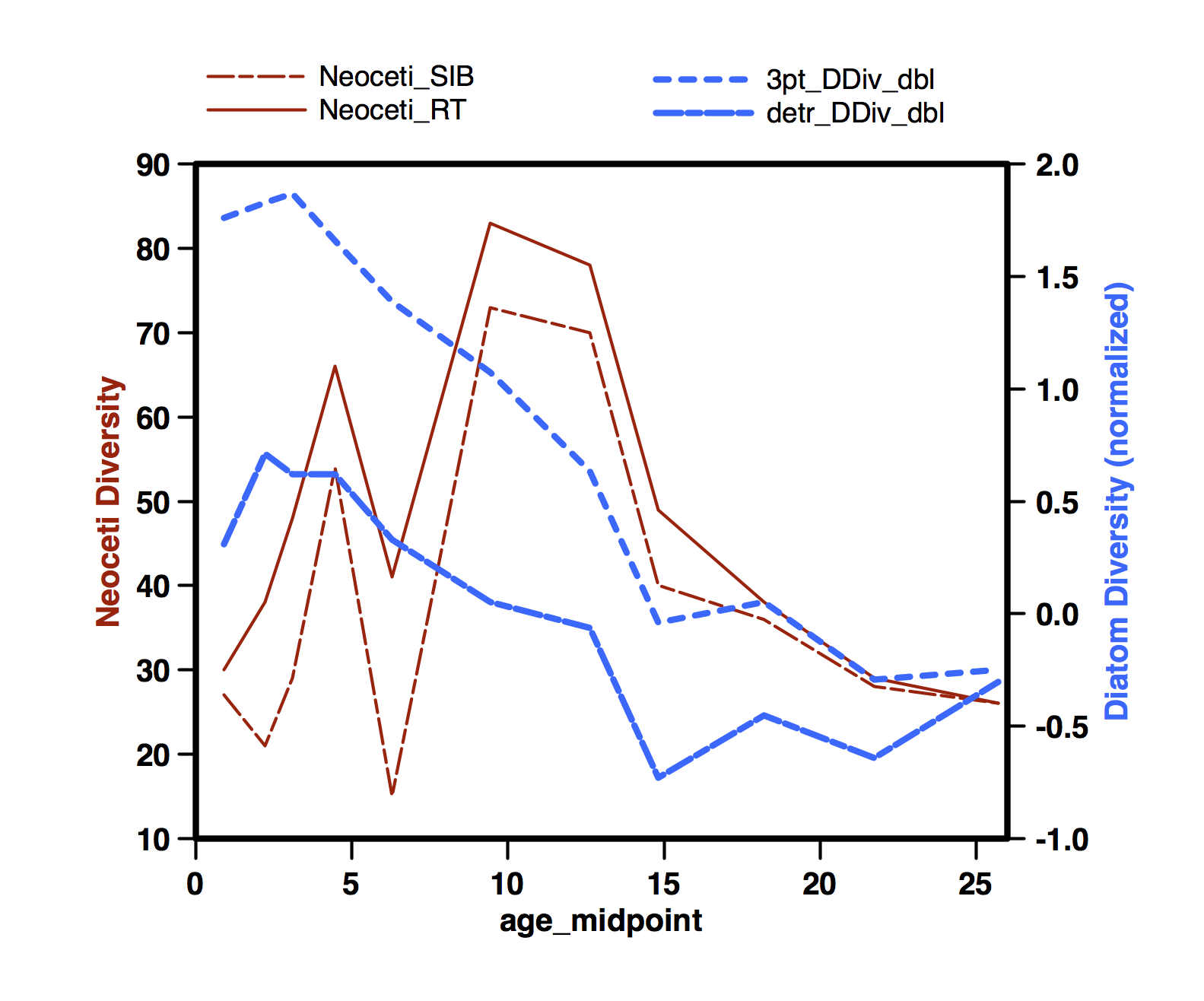

Supplement: Figure S3 — Comparison of species diversity in Neogene cetaceans (red: sampled in bin and range-through) to diatom diversity of this study (blue: 3-point moving average of raw data and Neogene portion of Cenozoic detrended values as reported in table ST2). Cetacean data from [61]. Age scale: [42]. (TIFF) [file pone.0084857.s003.tif]

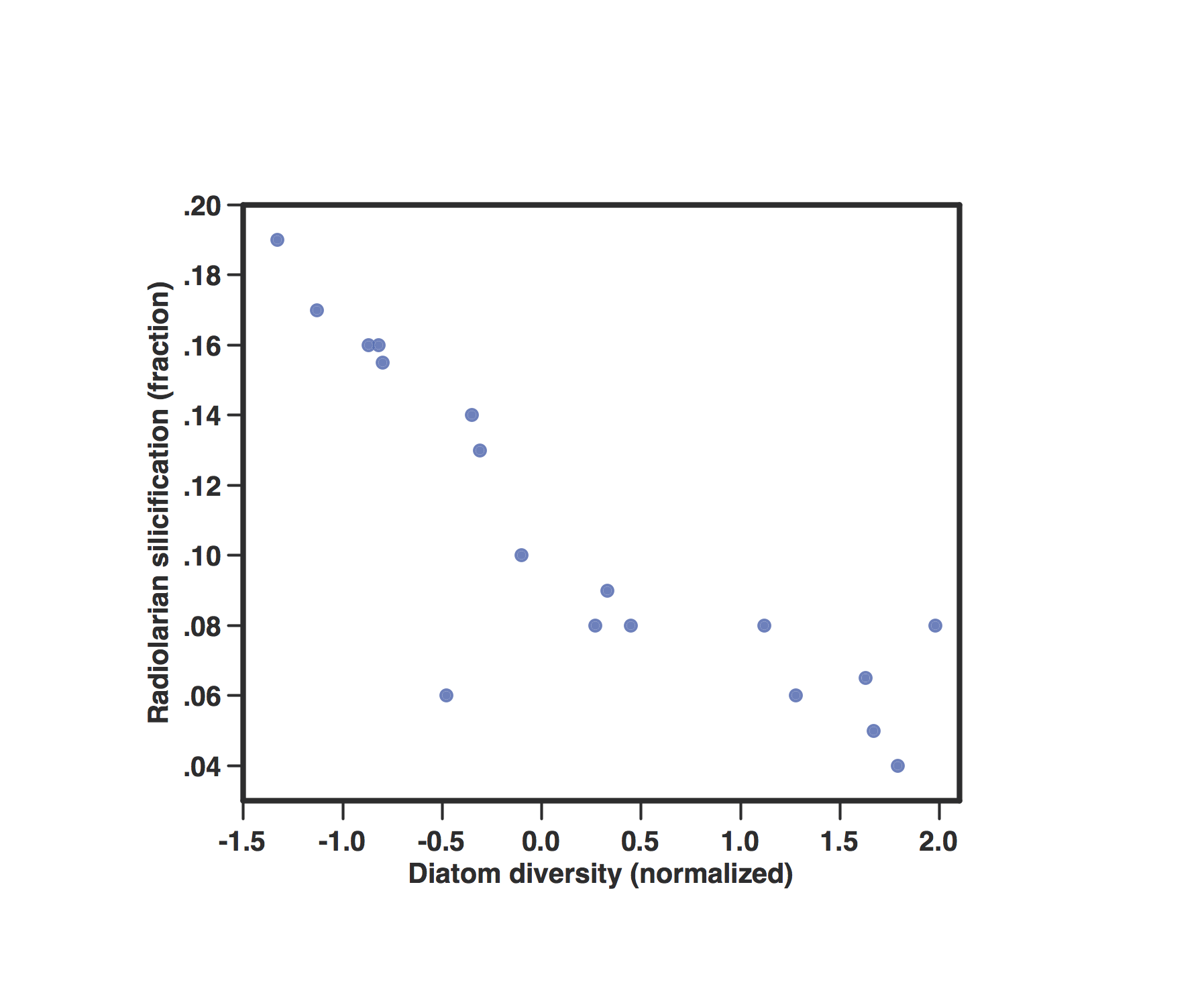

Supplement: Figure S4 — Diatom diversity (average of the three estimates for a bin, not smoothed) vs radiolarian shell silicification, from [48] . Silicification data binned to 1 my intervals, intervals with no silicification data excluded. Age scale: [43]. (TIFF) [file pone.0084857.s004.tif]

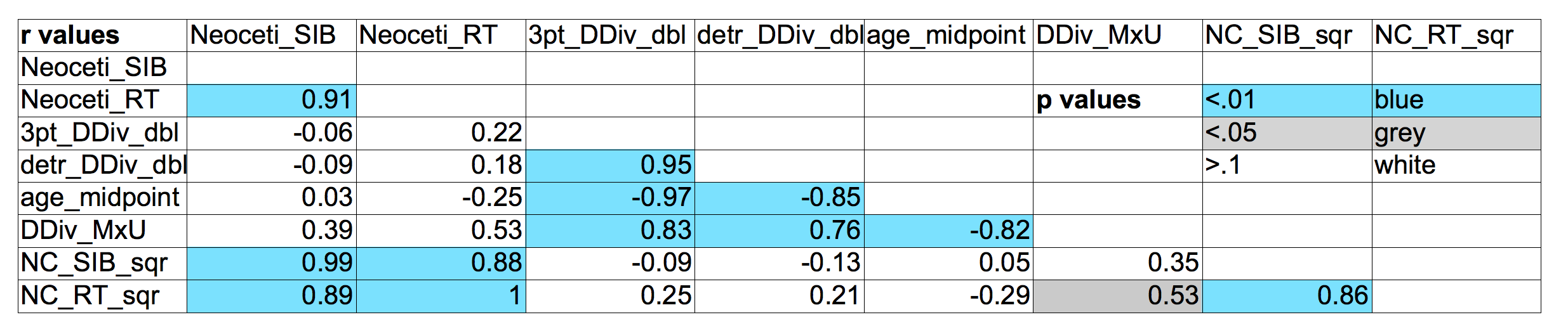

Supplement: Table S2 — Pearson correlation coefficient r and color coded p value intervals for diatom diversity, Neoceti diversity (from [61] ) and geologic age. (TIFF) [file pone.0084857.s006.tiff]
